# Interplay of Anisotropic Exchange Interactions and Single-Ion Anisotropy in Single-Chain Magnets Built from Ru/Os Cyanidometallates(III) and Mn(III) Complex

**DOI:** 10.3390/molecules28031516

**Published:** 2023-02-03

**Authors:** Vladimir S. Mironov, Eugenia V. Peresypkina, Kira E. Vostrikova

**Affiliations:** 1 Federal Scientific Research Centre of “Crystallography and Photonics”, Russian Academy of Sciences, Shubnikov Institute of Crystallography, Leninskiy Prospekt 59, 119333 Moscow, Russia; 2 Department of Chemistry and Pharmacy, Institute of Inorganic Chemistry, University of Regensburg, 31 Universitätsstr., 93053 Regensburg, Germany; 3 Nikolaev Institute of Inorganic Chemistry SB RAS, 3 Lavrentiev Avenue, 630090 Novosibirsk, Russia

**Keywords:** one-dimensional coordination polymer, ruthenium, osmium, single chain magnets, hexacyanometallates, Schiff base complexes, heterobimetallic complexes, magnetic anisotropy, anisotropic spin coupling

## Abstract

Two novel *1D* heterobimetallic compounds {[Mn^III^(SB^2+^)M^III^(CN)_6_]·4H_2_O}_n_ (SB^2+^ = N,N′-ethylenebis(5-trimethylammoniomethylsalicylideneiminate) based on orbitally degenerate cyanidometallates [Os^III^(CN)_6_]^3−^ (**1**) and [Ru^III^(CN)_6_]^3−^ (**2**) and Mn^III^ Schiff base complex were synthesized and characterized structurally and magnetically. Their crystal structures consist of electrically neutral, well-isolated chains composed of alternating [M^III^(CN)_6_]^3−^ anions and square planar [Mn^III^(SB^2+^)]^3+^ cations bridged by cyanide groups. These -ion magnetic anisotropy of Mn^III^ centers. These results indicate that the presence of compounds exhibit single-chain magnet (SCM) behavior with the energy barriers of Δ*τ_1_*/*k*_B_ = 73 K, Δ*τ_2_*/*k*_B_ = 41.5 K (**1**) and Δ*τ_1_*/*k*_B_ = 51 K, Δ*τ_2_* = 27 K (**2**). Blocking temperatures of *T*_B_ = 2.8, 2.1 K and magnetic hysteresis with coercive fields (at 1.8 K) of 8000, 1600 Oe were found for **1** and **2**, respectively. Theoretical analysis of the magnetic data reveals that their single-chain magnet behavior is a product of a complicated interplay of extremely anisotropic triaxial exchange interactions in M^III^(4d/5d)–CN–Mn^III^ fragments: −*J_x_S*_M_*^x^S*_Mn_*^x^*−*J_y_S*_M_*^y^S*_Mn_*^y^*−*J_z_S*_M_*^z^S*_Mn_*^z^*, with opposite sign of exchange parameters *J_x_* = −22, *J_y_* = +28, *J_z_* = −26 cm^−1^ and *J_x_* = −18, *J_y_* = +20, *J_z_* = −18 cm^−1^ in **1** and **2**, respectively) and single orbitally degenerate [Os^III^(CN)_6_]^3−^ and [Ru^III^(CN)_6_]^3−^ spin units with unquenched orbital angular momentum in the chain compounds **1** and **2** leads to a peculiar regime of slow magnetic relaxation, which is beyond the scope of the conventional Glaubers’s *1D* Ising model and anisotropic Heisenberg model.

## 1. Introduction

Over the past three decades, low-dimensional (LD), magnetically bistable coordination compounds featuring slow magnetic relaxation and blocking of magnetization at the molecular level [1,2,3,4,5,6,7,8] have attracted immense research interest inspired by their unique properties relevant to important future applications [9,10,11,12,13,14]. Depending on the dimensionality of the compounds, the magnetic systems are categorized into single-molecule magnets (SMMs, *0D* compounds) [15,16,17,18,19,20,21] and single-chain magnets (SCMs, *1D* compounds) [21,22,23,24,25,26,27]. Temperature-dependent magnetic dynamics of SMMs and SCMs is defined by the energy barrier for magnetization reversal, *U*_eff_, and blocking temperature *T*_b_, below which the magnetization is blocked and retained for a long period of time [16,23,26,28,29,30]. However, the mechanism underlying the slow relaxation and the origin of the energy barrier in *0D* and *1D* magnetic systems is different. In SMMs, negative uniaxial magnetic anisotropy (*D* < 0), together with high-spin ground state (*S*), is responsible for a double-well potential with the energy barrier *U*_eff_ = Δ = |*D*|*S*^2^ for integer *S* and |*D*|(*S*^2^−1/4) for half-integer *S* [1,3,28]. In SCMs, the barrier contains an additional energy term, Δ_ξ_, resulting from exchange coupling (*J*) between the magnetic ions, *U*_eff_ = *k*Δ_ξ_ + Δ, where *k* = 1 for finite or 2 for infinite chain [24,26,27,31,32]. In fact, Δ_ξ_ corresponds to the creation energy of the domain wall in the spin chain; it refers to a correlation energy. In the original Glauber’s model published back in 1963, for ferromagnetic Ising spin chain (*S*), the correlation energy is Δ_ξ_ = 4|*J*|*S*^2^ [33]. In the alternative model, SCM is described by a Heisenberg chain of spins with uniaxial single-ion magnetic anisotropy *D* and Δ_ξ_ = 4|*J*|*S*^2^ in the Ising limit (at |*D*/*J*| > 4/3) or Δ_ξ_ = 4*S*^2^(|*JD*|)^1/2^ in the Heisenberg limit (|*D*| << |*J*|) [34,35,36,37,38]. Basically, SCMs can be regarded as the *1D* analogs of SMMs, which are often thought to be more promising for rising the energy barrier as follows from an additional term Δ_ξ_ in *U*_eff_ [22,26,39].

Since the discovery of the first SCM in 2001 [22], a huge variety of SCMs with diverse architecture and magnetic properties have been synthesized using various spin carriers and bridging ligands [40,41,42,43,44,45]. However, most of the SCMs exhibit rather low spin reversal barriers (typically, *U*_eff_/*k*_B_ < 100 K) and blocking temperatures *T*_b_ within a few Kelvin. The record barrier of ca. 400 K and blocking temperature of 14 K are held by Co(II)-radical SCMs [25,46]. Among the cyanido-bridged *1D* systems, the largest barrier of 253 K has been reported for a Co^II^-[W^V^(CN)_8_]^3−^ bimetallic double-chain compounds [47]. To achieve high values of *U*_eff_ and *T*_b_, the building spin units in the chains must provide high magnetic anisotropy, strong intrachain magnetic coupling, and weak interchain interactions. Hence, for the rational molecular design of high-performance SCMs, the proper choice of magnetically anisotropic building units and tuning their magnetic interactions with spin carriers are crucial.

It is well recognized now that maximum magnetic anisotropy is ensured by molecular synthons with unquenched first-order orbital angular momentum: lanthanide (*4f*) [17,18,19] and actinide (*5f*) ions [48] as well as the special orbitally degenerate transition metal complexes [42,43,49,50,51,52,53,54,55,56,57]. In this regard, lanthanide ions (Ln) are especially appealing due to their large unquenched orbital angular momentum (L) and strong spin–orbit coupling (SOC), which—in concert with the ligand field splitting of *4f*-electrons—produce very strong single-ion magnetic anisotropy [17,18,19,48]. Currently, lanthanide ions (especially heavy Tb^III^, Dy^III^, and Ho^III^ cations) are widely used for designing advanced SMMs [58,59,60,61], including recently reported Dy^III^-based complexes with record barriers (>1000 cm^−1^) [62,63] and blocking temperatures (up to 80 K) [30]. A number of Ln-based *1D* coordination compounds with slow magnetic relaxation were also reported in recent years [64,65,66,67,68,69]. However, the use of the Ln^3+^ ions for the SCMs design has so far led to less impressive results compared to SMMs mainly due to the absence of strong magnetic coupling of Ln^3+^ ions with other spin carriers caused by the core-like nature of *4f* electrons. Several synthetic strategies have been employed to develop improved Ln-SCMs [17,18,19,58,59,60,61], among which the bridging radical strategy seems to be the most promising. Its main idea is that the enhanced intrachain Ln-radical spin coupling combined with the strong single-ion anisotropy of the Ln^3+^ ions (especially when it has a uniaxial Ising-type character) should ultimately lead to high-performance SCMs [70,71].

Among *3d* metal anisotropic building blocks, orbitally degenerate six-coordinated Co^II^ complexes are particularly popular for assembling magnetic chains [50,51,53,54,56,57]. Several Co^II^ complexes with less common coordination environments, such as pentagonal–bipyramidal complexes [72,73], were also used in the SCMs [54]. Remarkably, in low-dimensional (LD) transition metal complexes, both record barriers were achieved for the Co^II^-based compounds, *U*_eff_ = 594 K (413 cm^−1^) for SMMs [74] and 400 K for SCMs [25,46].

Octahedral hexacyanidoferrate(III) anion has been employed in many heterometallic chain compounds as a molecular linker between various spin carries [75,76,77,78,79,80]. First-order unquenched orbital momentum of the [Fe^III^(CN)_6_]^3−^ complex results in strong magnetic anisotropy of spin chains, which is imposed by sufficiently high anisotropic exchange interactions in the Fe^III^-CN-M-CN linkages, the latter having been explored in detail both experimentally [81,82] and theoretically [78,79,80]. Several magnetic chain compounds containing related orbitally degenerate metalloligands [Mn^III^(CN)_6_]^3−^ featuring slow magnetic relaxation were reported [83,84,85]. The physical mechanism of their SCM behavior is more complicated due to the nonmagnetic character of the singlet ground state of [Mn^III^(CN)_6_]^3−^ resulting from antiparallel coupling of the orbital (*L* = 1) and spin (*S* = 1) angular momentums, which cancel each other [85].

In recent years, efficient strategies to incorporate highly anisotropic *4d* and *5d* complexes into SMMs/SCMs have been actively developed [52,86,87,88,89,90,91,92,93,94,95,96,97,98,99,100]. Heavy transition metals offer exciting opportunities to rise the *U*_eff_ and *T_b_* parameters since the high energy and diffuse *4d* and *5d* orbitals provide stronger spin coupling as compared to *3d* orbitals of the first-raw transition metal complexes [86]. Another important advantage is in considerably stronger spin-orbit spin coupling of *4d* and *5d* electrons favoring enhanced magnetic anisotropy [86]. Numerous *0D* and *1D* molecular magnetic systems involving *4d* and *5d* metals were reported in the past decade [52,86,87,88,89,93,94,95,96,97,98,99,100,101]. However, in most cases, these SMMs and SCMs contain spin-only (nondegenerate) *4d* and *5d* complexes with enhanced second-order magnetic anisotropy. In particular, many heterometallic coordination compounds were based on Nb^IV^, Mo^V^, and W^V^ octacyanidometallates [90,91,93,94]. A number of high-spin (*S* = 3/2) *4d* and *5d* building units with strong zero-field splitting (ZFS) were employed in SMMs and SCMs: [Mo^III^(CN)_6_]^4−^ [95,96], [Re^IV^Cl_4_(CN)_2_]^2−^ [97,98,99], [Re^IV^(CN)_6_]^3−^ [101] and [Re^IV^F_6_]^2−^ [100]. Low-spin complexes [Ru^III^(acac)_2_(CN)_2_]^−^ [87], [M^III^(salen)(CN)_2_]^−^ (M = Ru [88], Os [89]) and other related systems are also popular in assembling SMMs and SCMs.

Given that magnetic anisotropy correlates with the value of L, orbitally degenerate *4d* and *5d* complexes with unquenched L (first-order compared to the ZFS of *3d*) are expected to be the most beneficial in reaching maximal magnetic anisotropy. Currently, they are mainly presented by the pentagonal–bipyramidal heptacyanidometallates [Mo^III^(CN)_7_]^4−^ [102,103] and [Re^IV^(CN)_7_]^3−^ [101,104,105] and octahedral hexacyanidometalles [Ru^III^(CN)_6_]^3−^ [106,107] and [Os^III^(CN)_6_]^3−^ [107,108,109]. Previous theoretical calculations showed that these low-spin orbitally degenerate (*S* = 1/2) complexes exhibit highly anisotropic spin coupling with connected spin carriers [110,111]. This is potentially helpful in the development of high-performance SMMs [112,113,114]. Incorporation of these molecular synthons into heterometallic coordination compounds has already led to obtaining of SMMs [115,116,117,118,119,120,121], SCMs [122,123,124] and extended polymer structures with unusual magnetic behavior [49,92,125,126,127].

In this context, a synthesis of the first heterobimetallic magnetic chain compound involving orbitally degenerate [Os^III^(CN)_6_]^3−^ complex and high-spin Mn^III^ Schiff-base complex [128], which exhibits distinct SCM behavior with enhanced *U*_eff_ and *T*_b_ parameters, is worth mentioning. This system is of particular interest for understanding the underlying physical mechanism of slow magnetic relaxation in a magnetic chain with highly anisotropic non-Ising spin coupling. Theoretical analysis for the discrete [Os^III^(CN)_6_]^3−^-based trinuclear clusters with similar local cyanide-bridging topology [119,121] showed that the spin coupling in the Os^III^–CN–Mn^III^ fragments is described by an extremely anisotropic triaxial spin Hamiltonian −*J_x_S*_M_*^x^S*_Mn_*^x^*−*J_y_S*_M_*^y^S*_Mn_*^y^*−*J_z_S*_M_*^z^S*_Mn_*^z^* with opposite signs of the exchange parameters, such as *J_x_* = −18, *J_y_* = +35, *J_z_* = −33 cm^−1^ in the Mn^III^_2_Os^III^ cluster [119]. This points at a special regime of magnetic relaxation in the {NC–Os^III^–CN–Mn^III^–NC–}_n_ chains resulting from a complicated interplay of highly anisotropic non-Ising exchange interactions and single-ion ZFS anisotropy of Mn^III^ ions, which is even more sophisticated due to the noncollinear orientation of the local magnetic axes. Obviously, such a scenario is the subject of new magnetic physics, which can be considered neither within the existing Ising theory nor within the anisotropic Heisenberg model.

**Scheme 1:**
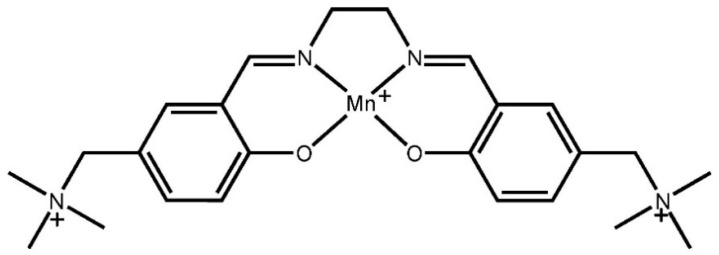
Molecular structure of triply charged *salen*-type [Mn(SB^2+^)]^3+^, SB^2+^ = N,N′-ethylenebis(5-trimethylammoniomethylsalicylideneiminate.

Based on these considerations, we prepared new heterometallic chain compounds involving [M^III^(CN)_6_]^3−^ metalloligand and Mn^III^ Schiff base cation (Scheme 1). In this study, we report on the synthesis, structure and magnetic properties of two novel isostructural bimetallic cyano-bridged chain compounds, {[Mn^III^(SB^2+^)M^III^(CN)_6_]·4H_2_O}_n_ based on orbitally degenerate [Os^III^(CN)_6_]^3−^ (**1**) or [Ru^III^(CN)_6_]^3−^ (**2**) hexacyanide and Mn^III^ Schiff base complex. Their crystal structure is built up of neutral, well-isolated chains composed of alternating [(Ru^III^/Os^III^)CN)_6_]^3−^ anionic complexes and square planar [Mn^III^(SB^2+^)]^3+^ cationic complexes. We present the results of static and dynamic magnetic measurements and a detailed theoretical interpretation based on the anisotropic spin coupling model. The presence of orbitally degenerate magnetic units with unquenched L is shown to lead to a peculiar regime of magnetic relaxation in the chain compounds **1** and **2** that goes far beyond the usual Ising and anisotropic Heisenberg models.

## 2. Results and Discussion

### 2.1. Synthetic Approach

Describing an approach chosen for the preparation of the new SCMs based on hexacyanidometallates as metalloligands, we would like point out that no SCM containing [Ru^III^(CN)_6_]^3−^ synthon has been obtained so far. The reason for this lies in the instability of hexacyanidoruthenate(III) anion in solution during slow-diffusion crystallization of heterobimetallic assemblies, unlike its iron and osmium congeners. With the latter, the anionic *1D* polymers [Mn^III^acacen(Fe^III^/Os^III^)(CN)_6_]^2−^ exhibiting SCMs properties were successfully obtained and studied [78,128], whereas for [Fe^III^(CN)_5_NO]^2−^ and [Re^IV^(CN)_7_]^3−^ *0D÷3D,* assemblies with Mn(III) complexes were obtained depending on synthetic conditions [49,92,101,127,129,130]. In such cases, the only way to obtain low-dimensional heterometallic complexes is to create conditions in which electroneutrality is a driving force of self-assembly. In order to guarantee a 1:1 stoichiometry in a chain or binuclear compound, identically charged counterions must be used as precursors. For cyanidometallates, this approach has been successfully developed and applied to the synthesis of neutral SCMs {[Mn^III^(SB^2+^)M^III^(CN)_6_]·4H_2_O}_n_ (M^III^ = Fe, Mn, Cr) [77] and {[Mn(SB^2+^)W(CN)_8_]·8H_2_O·MeCN}_n_ (**3**) [131] using the previously studied tricationic Schiff base complex [Mn(SB^2+^)(H_2_O)_2_]^3+^ [132]. In addition, the binuclear species [Mn^III^(SB^2+^)W^V^(CN)_8_]·4H_2_O·MeCN (**4**) and [Mn^III^(^Me^SB^2+^)M^III^(CN)_6_]·7H_2_O·MeCN (M^III^ = Fe, Mn, Cr) were obtained [131,133].

Owing to the low stability of the [Ru(CN)_6_]^3−^ anion in solution [106,107,134] compound **2** was obtained through rapid precipitation of the coordination polymer by mixing solutions, a procedure earlier used for the preparation of **3** [131]. Chain **1** was synthesized using a process similar to one described in Reference [77]. A layering of H_2_O:MeCN solutions containing [Mn(SB^2+^)(H_2_O)_2_](ClO_4_)_3_·H_2_O and (Ph_4_P)_3_[Os(CN)_6_] in a 1:1 ratio after a few days gave fern-like dark crystals slightly powdered by a white precipitation of Ph_4_PClO_4_, which was removed via washing in a few milliliters of acetonitrile. The data of IR, CHN analysis, and powder XRD confirmed the good quality and purity of the samples.

### 2.2. Crystal Structure

**Figure 1:**
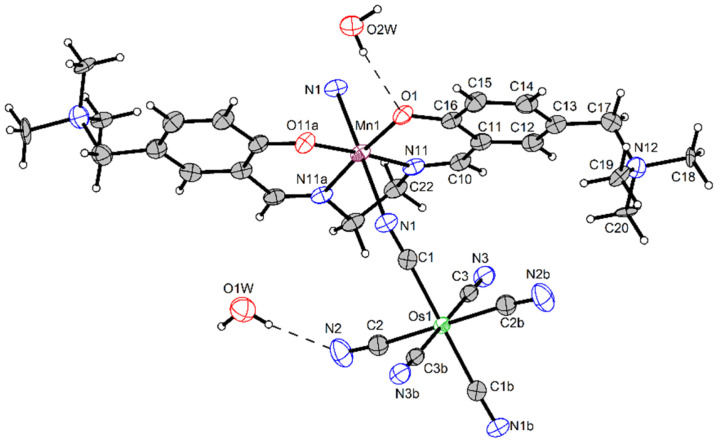
ORTEP view of the structure of **1**, showing the atom-labeling scheme. Displacement ellipsoids are drawn at the 50% probability level.

**Figure 2:**
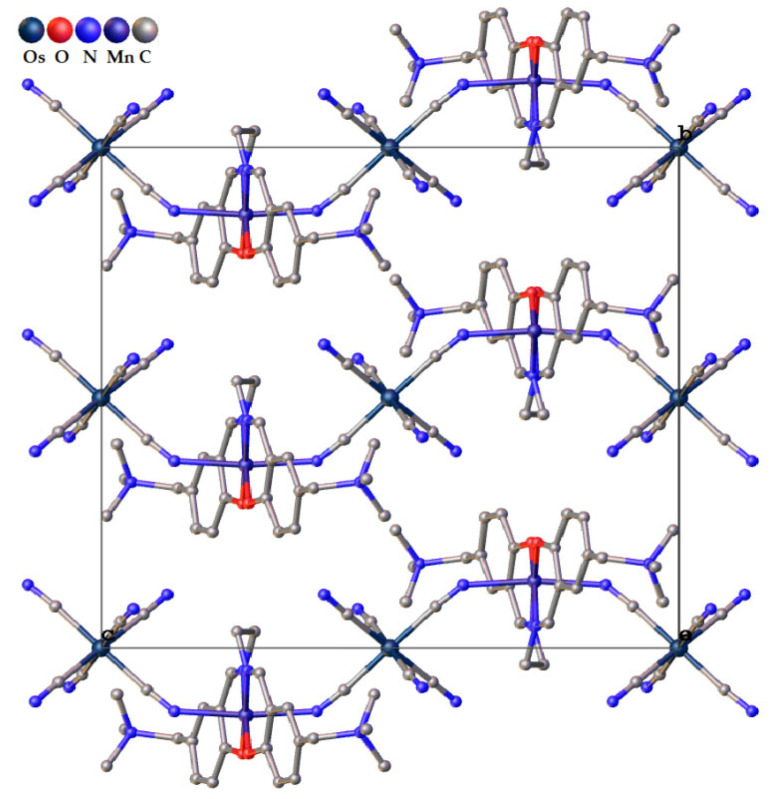
View of the chain motif of **1** projected along *a* axis. Hydrogen atoms and interstitial water molecules have been omitted for clarity. The axes denote overlap with atoms.

For **1**, we were able to grow crystals suitable for single-crystal X-ray structure analysis. This study has shown that Os-Mn polymer is isostructural to its earlier studied congener [Mn(SB^2+^)Fe^III^(CN)_6_]·4H_2_O [77], and the crystal unit cell parameters of **1** determined at 110 K are a = 11.2510(1), b = 16.7747(2), and c = 18.6078(2) Å β = 96.53(6)°, space group P2/c (#15) (Table S1, see Supplementary Materials)). The asymmetric unit is shown in Figure 1. The view of the *1D* chain motif is presented in Figure 2. Selected geometric parameters for **1** compared to its Fe congener are listed in Table 1. More bond lengths and bond angles are presented in Table S2. The coordination environment of the Mn ion is an elongated tetragonal bipyramid because of the Jahn–Teller distortion. The 2O and 2N donor atoms of the SB^2+^ ligand in the basal plane of the pyramid form shorter bonds of 1.88–1.98 Å, while two N atoms of *trans*-disposed CN ligands form much longer Mn–N_CN_ bonds of 2.28 Å with an _NC_N–Mn–N_CN_ angle of 173.5°, which is larger than in Mn-Fe analog (Table 1). The Mn–N–C bond angles are much more acute than 180° and equal to 142.5°, being slightly less than 144.4° for the Fe-containing chain. In **1**, Os ion coordinates four terminal CN groups, forming two hydrogen bonds N2···O1W of 2.94 Å and two N3···O2W of 2.88 Å. The additional contacts O1···O2W of 2.94 Å and O1W···O2W of 2.85 Å connect the neighboring chains into an H-bonded *3D* structure (Figure S1).

### 2.3. Powder X-ray Diffraction Investigations

The powder samples for the neutral heterobimetallic (Mn-Ru/Os) 1D polymers obtained via precipitation are crystalline, and their XRD patterns correspond well to the simulated diffractogram of the Mn-Fe chain, with the exception of one Bragg reflection (−1, 1, 2) (see Figures S2 and S3). This peak was calculated for the Mn-Fe compound significantly contributed by iron centers at 2θ = 12.867° (d = 6.8745 Å), but it is not observed due to a very low intensity. However, as the atomic weight of the central cyanidometallate atom increases, the corresponding peak emerges in the powder diffraction pattern for Ru and Os chains (Figures S2 and S3 and Table S3). The PXRD patterns confirmed that all three *1D* coordination polymers containing hexacyanidometallates(III) of the iron group are isomorphic.

### 2.4. Magnetic Studies

#### 2.4.1. Static Magnetic Properties and Their Theoretical Analysis

**Figure 3:**
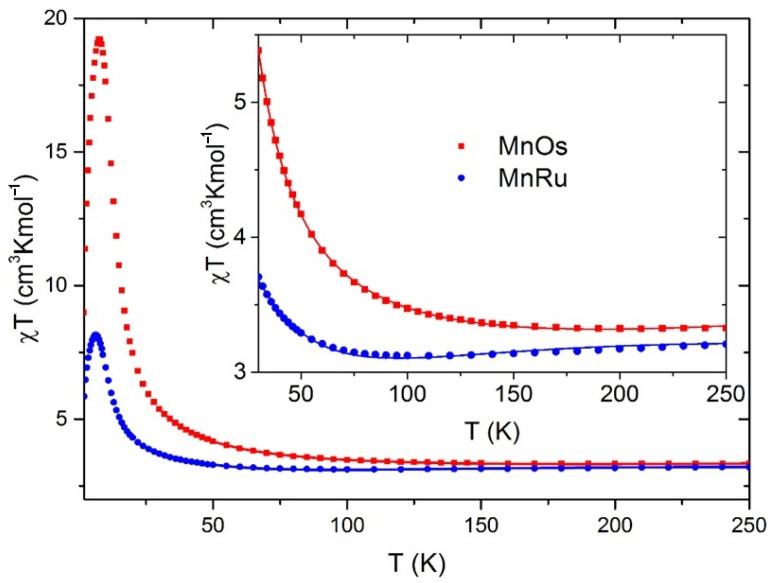
Magnetic susceptibility of **1** (red) and **2** (blue) in 1 kOe; insert: lines were fitted according to the Seiden’s model.

**Figure 4:**
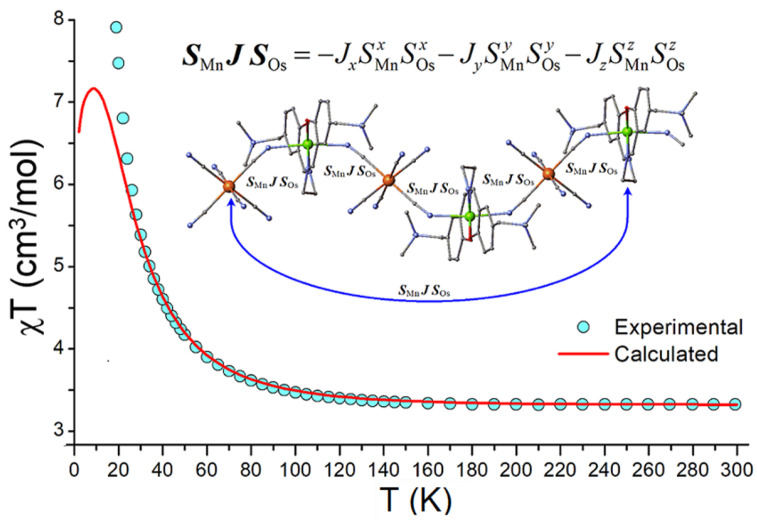
Experimental (blue circles) and simulated (solid red line) magnetic susceptibility *χT* of **1**. The *χT* curve was simulated with the spin Hamiltonian (2) involving anisotropic triaxial spin coupling ***S***_Mn_*J**S***_Os_ = −*J_x_*S_Mn_*^x^*S_Os_*^x^*−*J_y_*S_Mn_*^y^*S_Os_*^y^*−*J_z_*S_Mn_*^z^*S_Os_*^z^*. The best fit is obtained at the set of parameters *J_x_* = −22, *J_y_* = +28, *J_z_* = −26 cm^−1^, *g*_Mn_ = 2.02, *g*_Os_ =1.80, *D*_Mn_ = −3.6 cm^−1^. Calculations are performed for a six-membered fragment {Mn-Os}_3_ of the heterometallic chain of **1** with cyclic boundary conditions for the Mn-Os spin coupling, as shown in the inset.

The temperature dependences of the magnetic susceptibility measured at 1 kOe are shown in Figure 3 as the *χT* product. At 300 K, *χT* = 3.32 and 3.23 cm^3^K/mol for **1** and **2**, respectively, which agrees well with the Curie constant of 3.30 cm^3^K/mol expected for magnetically uncoupled Mn^III^ spin *S* = 2 with *g* = 2.0 and (Ru/Os)^III^ spin *S* = 1/2 with *g* = 1.8. As the temperature decreases starting from room temperature, the *χT* product of **2** slowly decreases, passing through a flat minimum around 100 K, and then rapidly rises at low temperatures to reach a sharp maximum of ~8 cm^3^K/mol at ~6 K. In contrast, *χT* of **1** increases monotonically, without a minimum, forming a much higher maximum of ~19 cm^3^ K/mol at ~8 K. It is noteworthy that the peak value of *χT* decreases with increasing field (Figure S4), showing saturation. A similar behavior of the *χT* function was observed in the closely related anionic chain compound (Ph_4_P)_2_[Mn^III^(acacen)Os^III^(CN)_6_] [128]. However, the magnetization of **1** and **2** does not saturate even at 50 *k*Oe, which is a signature of strong magnetic anisotropy. Below, in the current paper, the origin of the magnetic anisotropy of **1** and **2** is examined theoretically; our calculations reveal strong anisotropy of the *χT* product (Figure 4 and Figures S5 and S6).

The magnitude of the spin coupling constant *J* in heterometallic chains of compounds **1** and **2** can approximately be estimated in terms of an isotropic Heisenberg model for alternating spins S_Os/Ru_ = 1/2 and S_Mn_ = 2, which is described by the Hamiltonian (1):

(1) $$\hat{H}=-J\sum_{i}(S_{\mathrm{Mn}}^{i}+S_{\mathrm{Mn}}^{i+1})\cdot S_{\mathrm{Os}/\mathrm{Ru}}^{i}$$

A solution of this Hamiltonian, in the approximation that large spins *S*_Mn_ are treated classically, was obtained by Seiden as an analytical formula for susceptibility [135]. The only *J* value was adopted equally for all Ru-Mn or Os-Mn pairs and *g*_Ru/Os_ = 2 was fixed for both chains. The best fit to the magnetic data in the range of 30–300 K has resulted in and *g*_Mn_ = 1.76 and *J*/*k*_B_ = +25.4 K for **1** and *g*_Mn_ = 2.23, *J*/*k*_B_ = −62.8 K for **2** (inset in Figure 3). To improve the fit, an additional parameter *zJ’* was added in order to take into account interchain interactions, yielding and 0.51 K for **1**–1.39 K for **2**. The obtained values imply antiferromagnetic interactions within the chain for **2**, with weak ferromagnetic interchain coupling, while for **1**, the interactions within the chain are strong ferromagnetic, with weaker antiferromagnetic coupling between the chains.

These data indicate that the Seiden’s model results in unreliable and inconsistent magnetic parameters that are difficult to expect for the isostructural and isoelectronic chain compounds **1** and **2**. This is particularly apparent from the opposite sign of the exchange parameters (F in **1** and AF in **2**) and the large scatter in the effective *g*-factor of Mn^III^ ions. The basic reason behind these issues is that the isotropic Heisenberg model does not account for the anisotropic magnetic interactions associated with single-ion ZFS anisotropy of Mn^III^ ions and anisotropic exchange interactions of Os^III^ and Ru^III^ ions due to unquenched orbital angular momentum in the ground state. The origin of highly anisotropic exchange interactions in the Os^III^-CN-Mn^III^ and Ru^III^-CN-Mn^III^ exchange-coupled pairs was examined in detail in Ref. [119] for cyanide-bridged trinuclear clusters [Mn^III^_2_(5-Brsalen)_2_(MeOH)_2_M^III^(CN)_6_] (M = Os, Ru) whose structures are very close to the local structure of the chains in compounds **1** and **2**. The ground state of the Os^III^ ion in the octahedral ligand field of the [Os^III^(CN)_6_]^3−^ complex is an isotropic Kramers doublet *Г_7_* resulting from the spin orbit splitting of the ground orbital triplet *^2^T_2g_(5d^5^)*. The energy separation *ΔE* = 3/2*ζ_Os_* = 4500 cm^−1^ to the first excited state Г_8_ is determined by the spin orbit coupling constant (*ζ_Os_* ≈ 3000 cm^−1^). The Ru^III^ ion in complex [Ru (CN)_6_]^3−^ behaves similarly but with a smaller spin orbit coupling constant (*ζ_Ru_* ≈ 880 cm^−1^). It has been shown that the spin coupling between the ground Г_7_ state of Os^III^ (corresponding to the effective spin S = 1/2) and Mn^III^ ions (S = 2) is described by an anisotropic triaxial spin Hamiltonian, ***S***_Mn_*J**S***_Os_ = −*J_x_S*_Mn_*^x^S*_Os_*^x^*−*J_y_S*_Mn_*^y^S*_Os_*^y^*−*J_z_S*_Mn_*^z^S*_Os_*^z^*, with opposite sign of exchange parameters, *J_x_* = −18, *J_y_* = +35, *J_z_* = −33 cm^−1^ [119].

Hence, given close similarity in the molecular structure of trinuclear clusters [Mn^III^_2_(5-Brsalen)_2_(MeOH)_2_M^III^(CN)_6_] and chain compounds **1** and **2**, the same anisotropic spin Hamiltonian can be applied to the alternating heterometallic Os-Mn and Ru-Mn chains (2):

(2) $$\hat{H}=\sum_{<ij>}S_{Os(i)}JS_{\mathrm{Mn}(j)}+\sum_{j}S_{\mathrm{Mn}(j)}(T_{j}(\theta )D_{j}T{\left(\theta \right)}_{j}^{-1})S_{\mathrm{Mn}(j)}+\mu_{B}g_{\mathrm{Mn}}H\sum_{j}S_{\mathrm{Mn}(j)}+\mu_{B}g_{\mathrm{Os}}H\sum_{i}S_{\mathrm{Os}(i)}$$

where the sum <*ij*> runs over the neighboring Os(*i*) and Mn(*j*) cyanide-bridged exchange-coupled ions in the chain; the tensor of anisotropic spin coupling (***J***) has a three-axis structure, ***S***_Mn_***JS***_Os_ = −*J_x_S*_Mn_*^x^S*_Os_*^x^*−*J_y_S*_Mn_*^y^S*_Os_*^y^*−*J_z_S*_Mn_*^z^S*_Os_*^z^*. The ZFS ***D****_j_* tensors of the Mn^III^(*i*) ions are supposed to have the axial structure (with *D* < 0 and *E* = 0). However, each ***D****_j_* tensor of Mn^III^(*i*) ion is transformed by the *T_j_*(θ) rotation matrix, ***D****_j_*’ = *T_j_*(θ)***D****_j_T_j_*(θ)^−1^, specifying the noncollinear orientation (θ = ±37.5°) of these ZFS tensors in the bent structure of the {Mn-Os}*_n_* chain with respect to the local spin quantization axis *z* of the anisotropic exchange spin Hamiltonian ***S***_Mn_***JS***_Os_ (see inset in Figure 4).

Calculation of magnetic susceptibility *χT* using Equation (2) was performed for a finite six-membered chain {Os^III^-CN-Mn^III^-NC-}_3_, applying the cyclic boundary condition for the terminal Os^III^ and Mn^III^ spin centers as shown in Figure 4. The best fit to the experimental data for **1** is obtained at the set of parameters *J_x_* = −22, *J_y_* = +28, *J_z_* = −26 cm^−1^, *g*_Mn_ = 2.02, *g*_Os_ =1.80, and *D*_Mn_ = −3.6 cm^−1^. The simulated *χT* curve agrees well with the experimental data in the temperature range of 20–300 K. The divergence with the experimental data at low temperatures (below 20 K) is mostly due to the long-range spin correlation effects in an infinite chain, which cannot be reproduced in a finite-size chain employed in the calculations, Figure 4. The calculated anisotropic exchange parameters *J_x_*, *J_y_*, *J_z_* are well consistent with those obtained for discrete trinuclear Mn^III^_2_Os^III^ clusters, such as *J_x_* = −18, *J_y_* = +35, *J_z_* = −33 cm^−1^ [119] and *J_x_* = −23.5, *J_y_* = +32.0, *J_z_* = −25.9 cm^−1^ [121]. Similar calculations for the ruthenium chain compound **2** resulted in *J_x_* = −18, *J_y_* = +20, *J_z_* = −18 cm^−1^, *g*_Mn_= 2.00, *g*_Os_ = 1.80 and *D*_Mn_ = −4.0 cm^−1^ (Figure S6). Again, the calculated anisotropic exchange parameters are reasonably consistent with those obtained for the trinuclear Mn^III^_2_Ru^III^ complex, *J_x_* = −20, *J_y_* = +25, *J_z_* = −26 cm^−1^ [119].

#### 2.4.2. Magnetic Relaxation Parameters of 1 and 2 Derived from Static Magnetic Measurements

**Figure 5:**
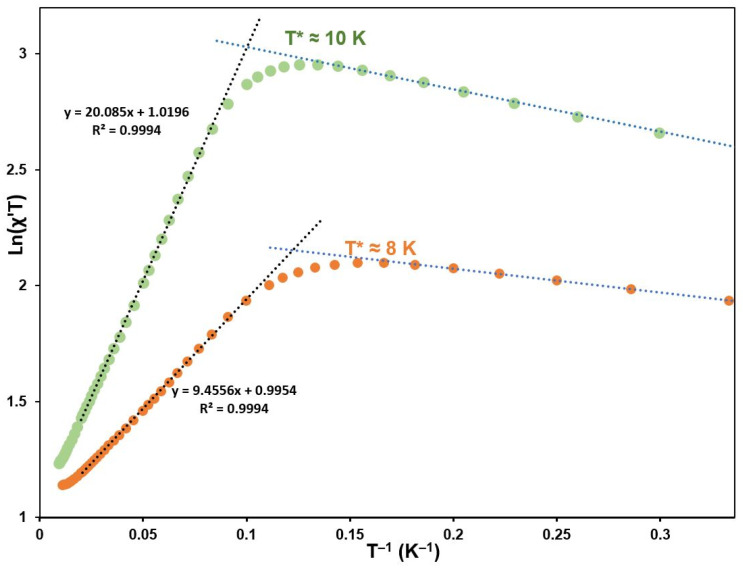
Magnetic *dc* susceptibility measured at one kOe for **1** (green) and **2** (orange), respectively. Straight dotted lines were fitted (see text). T* represents a crossover temperature.

Low-field *dc* measurements were used to determine the ∆_ξ_ parameter for **1** and **2**. Anisotropic 1D magnetic systems have a gap in the spin excitation energy spectrum, which leads to the susceptibility dependence *χT* ≈ *C_eff_Exp*(∆*_ξ_/T*) [26]. In this equation *C_eff_* is an effective Curie constant, which takes into account the averaging of anisotropic magnetic susceptibility in the powder sample. ∆_ξ_ designates an energy of the domain wall, which is the lowest excitation of the ground state in the chain of correlated spins. To estimate ∆_ξ_, the susceptibility data measured in 1 kOe were plotted as ln(*χT*) vs. *T*^−1^ (Figure 5). The linear part of the plot in the region from 10 to 30 K was used to obtain ∆_ξ_/*k*_B_ = 20.09 K and 9.45 K, and *C_eff_* = 2.77 and 2.71 cm^3^ K/mol for **1** and **2**, respectively. If the linear sections of the curves at the temperatures below 8 K will be drawn, the intersection of these straight lines with the linear parts of the plots between 10 and 30 K will give the crossover temperatures. The latter determine a crossover between infinite chain regime and finite chain regime in the relaxation dynamics. In our case, they are 10 and 8 K for **1** and **2** respectively.

For the real SCMs, below a certain temperature, the *χT*(1/*T*) dependence deviates from the exponential function and saturates (Figure 5)—even for a low applied field—due to the finite chain length caused by crystal imperfection [26]. This occurs when the rising correlation length surpasses an average chain length *n*. For **1**, it is visible below 8 K, where (*χT*)*_max_* = 22.1 cm^3^K/mol, and it is visible below 6 K for **2**, for which (*χT*)*_max_* = 8.56 cm^3^K/mol (data measured at 3 and 15 Oe, respectively). The average chain length estimated using the relation (*χT)_max_* = *nC*_eff_, gives *n* ≥ 7.94 nm for **1** and *n* ≥ 3.17 nm for **2**, which correspond to 16 Mn-Os and 6 Mn-Ru units, respectively (7.94 or 3.17 nm /5.166 Å). However, such an estimation of *n* should be treated with precaution because two other effects can also decrease the measured susceptibility in this temperature range: (a) possible antiferromagnetic inter-chain interaction and (b) demagnetization leading to a decrease in the measured *χ*. For this reason, the estimation given above is a lower limit of *n*.

#### 2.4.3. Dynamic Magnetic Properties

To examine the magnetic dynamics of **1** and **2**, the temperature-dependent and frequency-dependent *ac* susceptibilities were measured.

**Figure 6:**
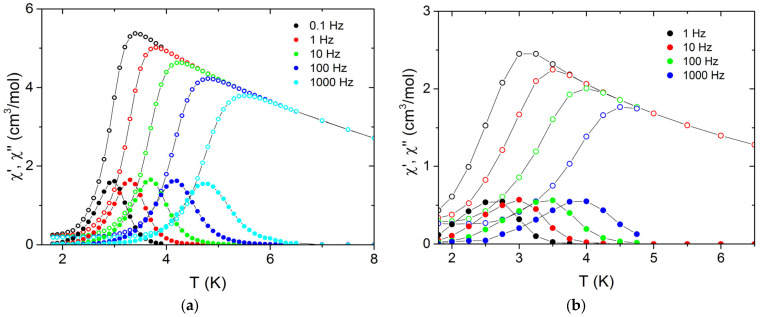
Real (empty dots) and imaginary (full dots) parts of *ac* susceptibility for **1** (**a**) and **2** (**b**) measured at different *ac* frequencies using *H*_ac_ = 3 Oe in zero *dc* field. Solid lines are to guide the eye.

Below 6 and 5 K for **1** and **2**, respectively, the *ac* susceptibility shows a distinction between different frequencies, indicating slow magnetization relaxation. In Figure 6, the temperature dependence of the *ac* susceptibility measured at zero *dc* field for different *ac* field frequencies is presented. The imaginary part of the *ac* susceptibility *χ*″(*T*) shows maxima below 5 and 6 K, shifting with the change of the *ac* drive field frequency υ, and retains the shape that is usual for a temperature induced relaxation process. The Mydosh parameter α, defined as the temperature shift of χ′(*T*) peak position on a decade of frequency Δ*T_m_*/[*T_m_*Δlog(υ)], remains around 0.10 in both cases. Such a value is above the range typical for spin glasses and is closer to the values for superparamagnets [136].

**Figure 7:**
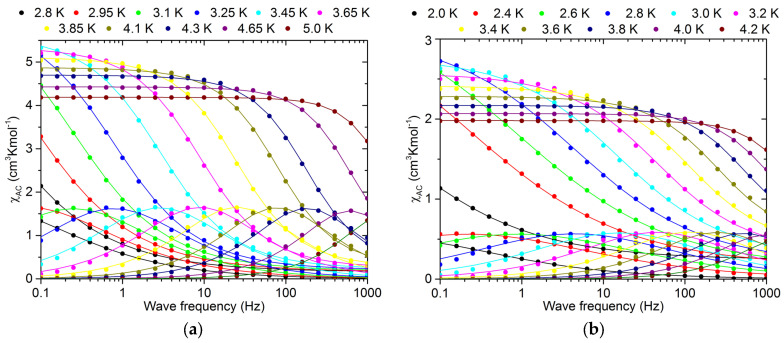
*ac* susceptibility measured for **1** (**a**) and **2** (**b**) at selected temperatures versus *ac* frequency. Solid lines were fitted simultaneously to *χ*’(*ν*) and *χ*’’(*ν*) curves using a generalized Debye relaxation model.

For a deeper understanding of the relaxation processes, the *ac* susceptibility was studied over the frequency range 0.1–1000 Hz at low temperatures. These data are presented in Figure 7. The frequency dependent susceptibility measured at constant temperatures was used to determine the relaxation time at each temperature. The generalized Debye relaxation model [137] was used to fit χ′(υ) and χ″(υ) simultaneously (Equation (3)) (solid lines in Figure 7).

(3) $$\chi =\chi \prime -i\chi \prime \prime =\chi_{\infty }+\frac{\chi_{0}-\chi_{\infty }}{1+{\left(i2\pi \nu \tau \right)}^{1-\alpha }}$$

At each temperature, the fitted parameters were χ_0_ and χ_∞_, the relaxation time *τ* and the parameter *α,* which describes the distribution of relaxation times. The values of *α* were in the range 0.11–0.58 for **1** and 0.09–0.47 for **2** (Tables S4 and S5), confirming the good quality of the sample and indicating the SCM nature of both compounds.

**Figure 8:**
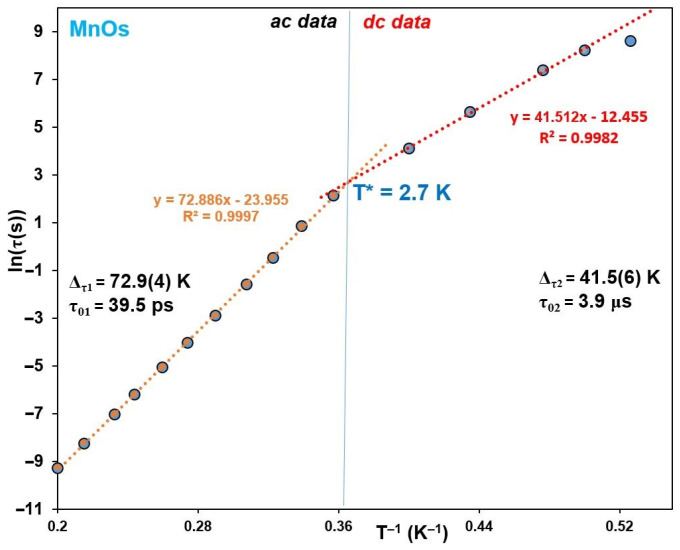
Relaxation time of **1** derived from the *ac* data (left) and time dependent *dc* magnetization (right). The dotted lines correspond to the fit according to the Arrhenius law: *τ = τ_01·_*exp*(*Δ*_τ1_/k_B_T)*. T* represents a crossover temperature.

The relaxation times in the temperature range from 2.0 to 5.0 K for **1** and 1.8 to 4.2 K for **2**, obtained from the *ac* (Figure 7) and *dc* (Figure S7) susceptibility analysis, are presented in Figure 8 and Figure S8. The dependence ln(*τ)–*(1/*T*) deviates from the straight line of the Arrhenius law. This is a feature of experimentally studied SCMs with finite chains, for which, below the crossover temperature T*, the probability of relaxation arising from the ends of chains becomes important, changing the relaxation barrier [138]. Above T*, where the correlation length ξ is lower than the average chain length *l* = *na*, the relaxation barrier is equal to Δ*τ_1_ =* Δ*_A_ + 2*Δ*ξ*, where Δ*_A_* is the anisotropy energy of a single spin unit. Below T*, the relaxation barrier is reduced and equal Δ*τ_2_ =* Δ*τ_1_ +* Δ*ξ* in the low temperature limit. The values of *Δτ_1_* and *Δτ*_2_ are usually obtained from two linear regions of the ln(*τ)–*(1/*T*) dependence much above—and much below—T*, respectively. To obtain both relaxation barriers using all data points also close to T*, we used the relation derived by Luscombe et al. for the finite Ising chain [138]. The finite length *l* of the chain shortens the relaxation time by the factor *f*(*l*/*ξ*):

*τ* = *τ_01_* {exp(Δ*_τ1_*/*k_B_*)} *f(l/ξ)*, (4)

where *f(x) = (1 + w^2^/x^2^)^−1^*, and *w* is the solution of the equation,

(5) $$w\tan\left(\frac{w}{2}\right)=x,\;\mathrm{in}\;\mathrm{the}\;[0,\pi ]\;\mathrm{range}.$$

Together with temperature dependence of the correlation length $\xi =\frac{a}{2}\exp\left(\frac{\Delta \xi }{kB\;T}\right)$ Equation (4) allows the calculation of *τ*(*T*) as the function of parameters Δ*ξ*, Δ*_τ1_*, *τ_01_*, and *l/a*. Equation (5) was solved numerically using the bisection method for *x* > 10^−3^, while the approximation *w* = (2*x*)^1/2^ was used for small values of *x* < 10^−3^.

It is worth noting that the crossover temperatures T* of 2.7 and 2.3 K for **1** and **2**, respectively—which were obtained from the intersection of the linear lines in Figure 8 and Figure S8—are very close to those of 3.7 and 2.65 K, respectively, calculated from ln(*χT*) vs. *T*^−1^ dependencies for the susceptibility data collected at the 0 *dc* and 3 Oe *ac* fields at 1 Hz below 9 and 4.2 K for **1** and **2**, respectively (Figure S9). Moreover, they are in good agreement with the magnetization blocking temperatures *T_B_* ≈ 2.8 and 2.1 K found when registering hysteresis at different temperatures see Figure S10.

The appearance of slow relaxations in **1** and **2** was also confirmed by presence of the hysteresis loops opens below 2.8 and 2.1 K respectively. Their coercive field grows with decreasing temperature. There are no additional steps on the *M*(*H*) curves (Figure S10). Such a behavior is expected for SCMs, contrary to SMMs, where the quantum tunneling leads to a faster relaxation at the specific fields. Again, this effect is typically observed for SCMs [77].

### 2.5. Comparison of SCM Parameters of 1, 2 and [Mn(SB^2+^)Fe(CN)_6_]·4H_2_O (**3**)

The parameters related to SCM behavior of **1** and **2** are summarized in Table 2 and compared with those of **3** [77].

For the chain **1**, the total energy barrier for an infinite chain is Δ*τ_1_ =* Δ*_A_ + 2*Δ*_ξ_* = 73 K at high temperature (Figure 8). However, at low temperature for a finite size chain regime, Δ*τ_2_ =* Δ*_A_ +* Δ*_ξ_* = 41.5 K, giving Δ*_ξ_* = 31.5 K, which is considerably larger than Δ*_ξ_* = 20.09 K obtained from the ln(*χT*) vs. *T*^−1^ plot (Figure 5). On the other hand, the intrinsic anisotropic barrier Δ*_A_* = Δ*τ_1_* − 2Δ*_ξ_* = 10 K obtained from the plot in Figure 8 is much smaller than Δ*_A_* = Δ*τ_1_*−2Δ*_ξ_* = 32.82 K, calculated with Δ*_ξ_* = 20.09, resulting from the ln(*χT*) vs. *T*^−1^ dependence. This indicates that the SCM relaxation mechanism in **1** cannot be described in the frame of the traditional anisotropic Heisenberg SCM model or Glauber model. The reason lies in the interplay of two independent sources of magnetic anisotropy, i.e., single-ion ZFS anisotropy of Mn^III^ ions and strong three-axis exchange anisotropy in the Os-CN-Mn linkages. The same also applies to the ruthenium chain of compound **2**, and quantitative estimates of its SCM parameters Δ*_A_* and Δ*_ξ_* made from the data of Figure S8 are presented in Table 2. Similar unconventional SCM behavior was previously reported for Mn^II^(H_2_Dapsc)-Fe^III^(CN)_6_ chain complex based on [Fe^III^(CN)_6_]^3−^ units with unquenched orbital angular momentum featuring highly anisotropic spin coupling [80].

## 3. Conclusions

Two novel heterometallic 1D coordination polymers {[Mn^III^(SB^2+^)M^III^(CN)_6_]·4H_2_O}_n_ (SB^2+^ = N,N′-ethylenebis(5-trimethylammoniomethylsalicylideneiminate) based on orbitally degenerate cyanidometallates [Os^III^(CN)_6_]^3−^ (**1**) and [Ru^III^(CN)_6_]^3−^ (**2**) and Mn^III^ Schiff base complexes were synthesized and characterized structurally and magnetically. Their crystal structure consists of electrically neutral, well-isolated chains composed of alternating [M^III^(CN)_6_]^3−^ anions and square planar [Mn^III^(SB^2+^)]^3+^ cations bridged by cyanide metalloligands. *dc* and *ac* magnetic measurements reveal SCM behavior of the compounds with the energy barriers of Δ*τ_1_*/*k*_B_ = 73 K, Δ*τ_2_*/*k*_B_ = 41.5 K (**1**) and Δ*τ_1_*/*k*_B_ = 51 K, Δ*τ_2_*/*k*_B_ = 27 K (**2**). The blocking temperatures of T_B_ = 2.8 K, 2.1, and magnetic hysteresis with a coercive field (at 1.8 K) of 8000 Oe and 1600 were found for **1** and **2**, respectively. For the first time, it was shown that the SCM behavior of **1** and **2** originates from a complicated interplay of two independent sources of magnetic anisotropy in the ruthenium and osmium chains, namely, the single-ion ZFS magnetic anisotropy of Mn^III^ ions and the anisotropic three-axis spin coupling **S**_Mn_**JS**_M_ = −J_x_S_Mn_^x^S_M_^x^–J_y_S_Mn_^y^S_M_^y^–J_z_S_Mn_^z^S_M_^z^ in the cyanide-bridged M^III^-CN-Mn fragments. This is a result of the triple orbital degeneracy of the *^2^T_2g_*(*nd^5^*) ground state of [Os^III^(CN)_6_]^3−^ and [Ru^III^(CN)_6_]^3−^ complexes with unquenched orbital angular momentum. Anisotropic exchange parameters *J_x_*, *J_y_*, *J_z_* obtained from our theoretical calculations (*J_x_* = −22, *J_y_* = +28, *J_z_* = −26 cm^−1^ for osmium compound **1**) are remarkably consistent with those previously reported for discrete trinuclear Mn^III^_2_Os^III^ clusters with similar molecular structure, such as *J_x_* = −18, *J_y_* = +35, *J_z_* = −33 cm^−1^ [119] and *J_x_* = −23.5, *J_y_* = +32.0, *J_z_* = −25.9 cm^−1^ [121]. Our theoretical calculations and analysis of magnetic relaxation parameters Δ_A_ and Δ_ξ_ have distinctly showed that these new 1D coordination polymers **1** and **2** are SCMs beyond the Glauber model and the anisotropic Heisenberg SCM model.

## 4. Materials and Methods

All chemicals were of reagent grade and were used as purchased. The complex [Mn(SB^2+^)(H_2_O)_2_](ClO_4_)_3_·H_2_O [132] and hexacyanometallates [108] (Ph_4_P)_3_[Os(CN)_6_], (n-Bu_4_N)_3_[Ru(CN)_6_] and (n-Bu_4_N)_3_[Os(CN)_6_] were prepared according to procedures in the literature [55,128]. Elemental analyses were performed by means of the Euro-Vector 3000 analyzer (Eurovector, Redavalle, Italy). IR spectra were recorded using a Scimitar FTS 2000 spectrophotometer (Digilab LLC, Canton, MA, USA) (KBr pellets) and Nicolet 300 FT-IR spectrometer in reflectance mode (Thermo Electron Scientific Instruments LLC, Madison, WI, USA). Powder X-ray measurements were performed with CuKα radiation (*λ* = 1.5418 Å) with an Expert-Pro powder diffractometer (PANalytical Inc., Almelo, The Netherlands). Magnetic measurements were performed using the QD MPMS 5XL magnetometer (Quantum Design, Inc., San Diego, CA, USA). The magnetic signal of the sample holder and the diamagnetic correction of the sample were taken into account. A check for small ferromagnetic impurities was performed at room temperature. The powder sample was restrained in cyanoacrylate glue for low-temperature *ac* measurements.

**[Mn(SB^2+^)Os(CN)_6_]·4H_2_O** (**1**)**.** A solution of (Ph_4_P)_3_[Os(CN)_6_] (95 mg, 0.07 mmol) in H_2_O:CH_3_CN (1:3, 3 mL) was added dropwise to a solution of [Mn(SB^2+^)(H_2_O)_2_] (ClO_4_)_3_·H_2_O (47 mg, 0.07 mmol) in the same solvent (3 mL). A reaction mixture, permanently agitated, was heated gradually up to boiling and then cooled down. A dark olive powder was filtered off; washed a few times with the solvent, acetonitrile, and ether; and air-dried. Yield: 62.5 mg (95.3 %). Calculated for Mn(C_24_H_34_N_4_O_2_)(H_2_O)_5.4_Os(CN)_6_ (Ph_4_PClO_4_)_0.1_: C, 40.83; H, 4.95; N, 14.70; found: C, 40.7; H, 4.85; N, 14.7; IR (reflectance): $\tilde{\nu }$ = 2112, 2088 and 2049 (ν_C≡N_), 1632 cm^−1^ (ν_C=N_). The final products prepared starting from (Ph_4_P)_3_[Os(CN)_6_] are slightly contaminated by Ph_4_PClO_4_ because of its poor solubility in MeCN. For this reason, it is better to use (n-Bu_4_N)_3_[Os(CN)_6_] as a Os-precursor.

**[Mn(SB^2+^)Ru(CN)_6_]·4H_2_O** (**2**)**.** This compound was synthesized analogously to [Mn(SB^2+^)Os(CN)_6_]·4H_2_O with the difference that (n-Bu_4_N)_3_[Ru(CN)_6_] was used as a starting material, and a reaction mixture was not heated during precipitation of the final product. Yield 92 %. Calculated for C_28_H_28_MnN_10_O_2_Ru·4(H_2_O): C, 44.33; H, 5.46; N, 17.23; found: C, 44.7; H, 4.98; N, 17.5; IR (reflectance): ῡ = 2100, 2089 and 2048 (ν_C≡N_), 1630.8 cm^−1^ (ν_C=N_).

**Crystallographic Details.** Single crystals of **1** were obtained via slow diffusion of the starting solutions used for a bulk powder sample.

The single crystals covered by a drop of the oil were directly placed into a stream of cold nitrogen with the precentered goniometer head with CryoMount^®^ (Chelan County, WA, USA) and attached to the goniometer of a diffractometer. The data for **1** were collected on an Agilent Technologies Gemini diffractometer equipped with an Atlas^S2^ CCD detector and a CuKα microfocus source using 0.5° ω scans. The data processing was performed with the CrysAlis software package (CrysAlisPro 1.171.40.47a, Rigaku Oxford Diffraction, 2019). Empirical absorption correction was applied based on the equivalent reflections. The structure was solved by direct methods with *SHELXS* [139] and refined by full-matrix least squares method against *F^2^* in anisotropic approximation using the *SHELXL-2014/6* package (Shelx, Göttingen, Germany*)*. All non-hydrogen atoms were refined with anisotropic displacement parameters. Hydrogen atoms on the organic part were placed in idealized positions and refined isotropically according to the riding model. The hydrogen atoms of the water molecule were located from the electron density map and refined in a riding model (U_iso_(H) = 1.5 U_iso_(O)) with only one distance of 4 (O2w-H21) restrained to 0.82(2) Å. The residual electron density has to chemical meaning. Crystallographic data and further details of the diffraction experiments are given in Table S1.

**Theoretical calculations details.** Magnetic properties of chain compounds **1** and **2** were analyzed in terms of the anisotropic spin Hamiltonian in Equation (2), which involves contributions from the single-ion ZFS anisotropy of Mn^III^ ions and three-axis anisotropic spin coupling ***S***_M_***JS***_Mn_ = −*J_x_S*_M_*^x^S*_Mn_*^x^—J_y_S*_M_*^y^S*_Mn_*^y^—J_z_S*_M_*^z^S*_Mn_*^z^* in the M^III^-CN-Mn^III^ linkaged (M = Ru, Os). Computational details are described in Supporting Information. Specific calculations for **1** and **2** were performed with routines written by V.S. Mironov.

## Figures and Tables

**Table 1:** Selected geometric parameters for [Mn(SB^2+^)Os(CN)_6_]·4H_2_O (**1**).

	Mn-Os	Mn-Fe [77]
**Bond**	Distance (Å)	Distance (Å)
Mn1–O1 ^a^	1.884(2)	1.884(2)
Mn1–N11 ^a^	1.981(3)	1.983(3)
Mn1–N1 ^a^	2.281(3)	2.307(3)
M–C1 ^b^	2.050(4)	1.944(3)
M–C2 ^b^	2.060(4)	1.953(3)
M–C3 ^b^	2.064(4)	1.958(4)
C1–N1	1.158(5)	1.156(4)
C3–N3	1.154(5)	1.150(5)
C2–N2	1.150(5)	1.149(4)
**Hydrogen bond**		
N2···O1W	2.938	2.969
N3···O2W	2.884	2.912
O1···O2W	2.944	2.937
O1W···O2W	2.851	2.874
Angle	(°)	(°)
N1-Mn-N1	173.47(15)	170.80(9)
Mn-N1-C1	142.5(3)	144.4(3)

Symmetry codes: ^a^ −*x*, *y*, −1/2 − *z*; ^b^ − *x*, 1 − *y*, −*z*.

**Table 2:** Single chain magnet related parameters for **1**, **2**, and their **Fe** congener [77] (in K if not specified).

Chain	*J*/cm^−1^ *Jx, Jy, Jz/* cm^−1^	Δ *_ξ_/k_B_*	*α*	*τ_01_/s*	Δ *τ_1_/k_B_*	*τ_02_/s*	Δ *τ_2_/k_B_*	Δ *_ξ_* = (Δ*τ_1_*–Δ*τ_2_*)/*k_B_*	Δ *_A_* = (Δ*τ_2_*–Δ*_ξ_)*/*k_B_*	*T_b_*	H_A_*/kOe
Mn-Fe	3.128	14.1	0.26–0.33	5.2·10^−10^	32	8·10^−5^	16	16	≈0	1.6	108
Mn-Ru	−25.9, +28.8, −25.9	9.45	0.16–0.52	4.33·10^−10^	51	5.66·10^−6^	27	24	3	2.1	170
Mn-Os	−31.6, +40.3, −37.4	20.09	0.13–0.47	3.95·10^−6^	73	3.9·10^−3^	41.5	31.5	10	2.8	210

H_A_*—anisotropy field.

## Data Availability

The complete crystallographic data for **1** have been deposited with the Cambridge Crystallographic Data Centre under the reference numbers CCDC 1958305. These data can be obtained, free of charge, from the CCDC via www.ccdc.cam.ac.uk/structures (accessed on 29 September 2022).

## Supplementary Materials

The following supporting information can be downloaded at: https://www.mdpi.com/article/10.3390/molecules28031516/s1, Table S1: Experimental details for **1**; Table S2: Selected geometric parameters; Figure S1: Hydrogen bonding system in **1**; Table S3: Some crystallographic parameters for [Mn(SB^2−^)M(CN)_6_]·4H_2_O; Figure S2: Simulated (red) and experimental X-ray powder pattern for neutral Mn-Os chain polymer; Figure S3: Simulated and experimental X-ray powder patterns (red) for neutral Mn^III^-M^III^(CN)_6_ chain polymers (M^III^ = Fe, Ru, Os) with additional reflections (green rectangle) from (–1, 1, 2) plane consisted of metal atoms; Figure S4: Magnetic susceptibility times temperature vs. *T* at 1 kOe and lower fields for **1** in 3 Oe (left) and **2** in 15 Oe (right); Figure S5: Calculated components χ_x_, χ_y_, χ_z_ of magnetic susceptibility of the Mn-Os chain (**1**). Below 50 K, magnetic susceptibility is strongly anisotropic; Figure S6: Experimental (yellow circles) and simulated (solid blue line) magnetic susceptibility *χT* of **2**. The *χT* curve was simulated with the spin Hamiltonian (Equation (2)) involving anisotropic 3-axes spin coupling ***S***_Mn_***JS***_Ru_ = −*J_x_*S_Mn_*^x^*S_Ru_*^x^* −*J_y_*S_Mn_*^y^*S_Ru_*^y^* −*J_z_*S_Mn_*^z^*S_Ru_*^z^*. The best fit is obtained at the set of parameters *J_x_* = −18, *J_y_* = +20, *J_z_* = −18 cm^−1^, *g*_Mn_= 2.00, *g*_Ru_ =1.80, *D*_Mn_ = -4.0 cm^−1^. Calculations are performed for a six-membered fragment {Mn-Ru}_3_ of the heterometallic chain of **2** with cyclic boundary conditions for the Mn-Ru spin coupling, as shown in the inset; Table S4: Cole–Cole fits parameters for **1**; Table S5: Cole–Cole fits parameters for **2**; Figure S7: Time dependence of magnetization relaxation for **1** (left) and **2** (right) following the field change from 10 to 0 kOe at constant temperatures of 1.8 ÷ 2.5 K; Figure S8: Relaxation time of **2** derived from the *ac* data (left) and time dependent *dc* magnetization (right). The dotted lines correspond to the linear fit according to the Arrhenius law: *τ = τ_01_Exp*(Δ*_τ1_/k_B_T*); Figure S9: Crossover temperatures *T** obtained from the ln(*χT*) vs. *T*^−1^ dependencies for the susceptibility data collected at 0 *dc* and 3 Oe *ac* field at 1 Hz below 9 and 4.2 K for **1** (left) and **2** (right), respectively; Figure S10: Magnetization versus field for **1** (top) and **2** (bottom)—hysteresis loops. Solid lines are to guide the eye; Figure S11: Zero-field cooling/field cooling magnetic susceptibility vs. temperature for **1** and **2** in 15 Oe with a temperature sweep rate of 2 K/min; Figure S12: FTIR (ATR) spectra of **1** (top) and **2** (bottom).
