# Supplementary material for: Interplay of Anisotropic Exchange Interactions and Single-Ion Anisotropy in Single-Chain Magnets Built from Ru/Os Cyanidometallates(III) and Mn(III) Complex
Source: Molecules. 2023 Feb 3;28(3):1516. doi: 10.3390/molecules28031516 (PMC9921754; doi:10.3390/molecules28031516)
Supplement: Supplementary file 1 [file molecules-28-01516-s001.zip › molecules-2167625-supplementary.pdf]

Supporting Information

Interplay of Anisotropic Exchange Interactions and Single-Ion Anisotropy in Single-Chain Magnets Built from Ru/Os Cyanidometallates(III) and Mn(III) Complex

Vladimir S. Mironov [3]*, Eugenia V. Peresypkina [2] and Kira E. Vostrikova [1]*

Table S1. Experimental details for **1**.

Crystal data	
Chemical formula	$C_{28}H_{28}MnN_{10}O_2Os·4(H_2O)$
CCDC Code	CCDC-1958305
M_r	853.81
Crystal system, space group	Monoclinic, *C*2/*c*
Temperature (K)	123
a, *b*, *c* (Å)	11.25103 (11), 16.77467 (15), 18.60785 (18)
β (°), *V* (Å^3), *Z*	96.5256 (9), 3489.15 (6), 4
F(000), D_x (Mg m^{-3})	1692, 1.625
Radiation type	Cu *K*α
μ (mm^{-1})	10.17
Crystal size (mm)	0.41 × 0.25 × 0.20
Data collection	
Diffractometer	Xcalibur, Atlas[S2], Gemini ultra
Absorption correction	Multi-scan
T_{min}, T_{max}	0.083, 1.000
No. of measured, independent and observed [$I > 2\sigma(I)$] reflections	54476, 3098, 2915
R_{int}	0.051
$(\sin \theta/\lambda)_{max}$ (Å^{-1})	0.596
Range of *h*, *k*, *l*	*h* = -13→13, *k* = -19→19, *l* = -22→22
Refinement	
$R[F^2 > 2\sigma(F^2)]$, $wR(F^2)$, *S*	0.029, 0.080, 1.15
No. of reflections, No. of parameters, No. of restraints	3098, 231, 1
H-atom treatment	H atoms treated by a mixture of independent and constrained refinement
$\Delta\rangle_{max}$, $\Delta\rangle_{min}$ (e Å^{-3})	1.69, -0.74

Table S2. Selected geometric parameters (Å,º).

Os1—C1^b	2.050 (4)	N11—C10	1.284 (5)
Os1—C1	2.050 (4)	N11—C22	1.476 (5)
Os1—C2	2.060 (4)	N12—C20	1.483 (5)
Os1—C2^b	2.060 (4)	N12—C19	1.484 (6)
Os1—C3	2.064 (4)	N12—C18	1.497 (6)
Os1—C3^b	2.064 (4)	N12—C17	1.519 (5)
C1—N1	1.158 (5)	C10—C11	1.450 (5)
C3—N3	1.154 (5)	C11—C12	1.403 (5)
C2—N2	1.150 (5)	C11—C16	1.423 (5)
N1—Mn1	2.281 (3)	C12—C13	1.371 (6)
Mn1—O1^a	1.884 (2)	C13—C14	1.409 (5)
Mn1—O1	1.884 (2)	C13—C17	1.499 (5)
Mn1—N11^a	1.981 (3)	C14—C15	1.378 (5)
Mn1—N11	1.982 (3)	C15—C16	1.403 (5)
Mn1—N1^a	2.281 (3)	C22—C22^a	1.519 (8)
O1—C16	1.321 (4)		
C1^b—Os1—C1	180.0	O1—Mn1—N1^a	94.84 (10)
C1^b—Os1—C2	92.93 (14)	N11^a—Mn1—N1^a	86.08 (11)
C1—Os1—C2	87.08 (14)	N11—Mn1—N1^a	89.00 (11)
C1^b—Os1—C2^b	87.07 (14)	N1—Mn1—N1^a	173.47 (15)

C1—Os1—C2^{b}	92.92 (14)	C16—O1—Mn1	125.8 (2)
C2—Os1—C2^{b}	180.00 (19)	C10—N11—C22	120.9 (3)
C1^{b}—Os1—C3	91.00 (13)	C10—N11—Mn1	125.6 (2)
C1—Os1—C3	89.00 (13)	C22—N11—Mn1	113.3 (2)
C2—Os1—C3	90.12 (14)	C20—N12—C19	109.6 (4)
C2^{b}—Os1—C3	89.88 (14)	C20—N12—C18	108.0 (4)
C1^{b}—Os1—C3^{b}	89.00 (13)	C19—N12—C18	111.2 (4)
C1—Os1—C3^{b}	91.00 (13)	C20—N12—C17	110.9 (3)
C2—Os1—C3^{b}	89.88 (14)	C19—N12—C17	111.5 (3)
C2^{b}—Os1—C3^{b}	90.12 (14)	C18—N12—C17	105.6 (3)
C3—Os1—C3^{b}	180.00 (15)	N11—C10—C11	124.0 (3)
N1—C1—Os1	175.1 (3)	C12—C11—C16	119.5 (3)
N3—C3—Os1	178.8 (3)	C12—C11—C10	117.3 (3)
N2—C2—Os1	175.0 (4)	C16—C11—C10	123.2 (3)
C1—N1—Mn1	142.5 (3)	C13—C12—C11	121.9 (3)
O1^{a}—Mn1—O1	93.88 (14)	C12—C13—C14	118.5 (3)
O1^{a}—Mn1—N11^{a}	91.96 (11)	C12—C13—C17	121.0 (3)
O1—Mn1—N11^{a}	174.10 (11)	C14—C13—C17	120.3 (3)
O1^{a}—Mn1—N11	174.10 (11)	C15—C14—C13	121.0 (3)
O1—Mn1—N11	91.96 (11)	C14—C15—C16	121.2 (3)
N11^{a}—Mn1—N11	82.22 (17)	O1—C16—C15	118.5 (3)
O1^{a}—Mn1—N1	94.84 (10)	O1—C16—C11	123.4 (3)
O1—Mn1—N1	89.62 (11)	C15—C16—C11	118.0 (3)
N11^{a}—Mn1—N1	89.00 (11)	C13—C17—N12	115.3 (3)
N11—Mn1—N1	86.08 (11)	N11—C22—C22^{a}	106.9 (2)
O1^{a}—Mn1—N1^{a}	89.62 (11)		

Symmetry code(s): (a) -*x*, *y*, -1/2-*z*; (b) -*x*, 1-*y*, -*z*.

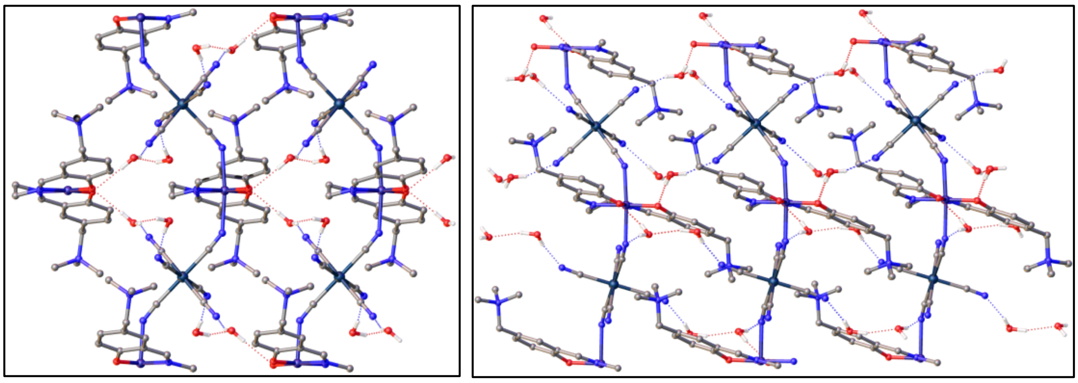

Figure S1. Hydrogen bonding system in **1**.

Table S3. Some crystallographic parameters for [Mn(SB^{2-})M(CN)$_6$]·4H$_2$O.

M	Conditions	Cell parameters P2/c; a, b, c (Å); β°	2θ	d-spacing,	h k l
Fe*	SCXRD, 93 K	11.236, 16.741, 18.457, 96.929	12.867	6.8745	-1 1 2
Ru	PXRD, RT	11.246, 16.743, 18.569	12.846	6.8857	-1 1 2
Os	SCXRD, 110 K	11.2510, 16.7747, 18.6078, 96.53	12.837	6.8907	-1 1 2

*H. Miyasaka, T. Madanbashi, A. Saitoh, N. Motokawa, R. Ishikawa,M. Yamashita, S. Bahr, W. Wernsdorfer, R. Clérac, *Chem. Eur. J.,* 2012, **18**, 3942.

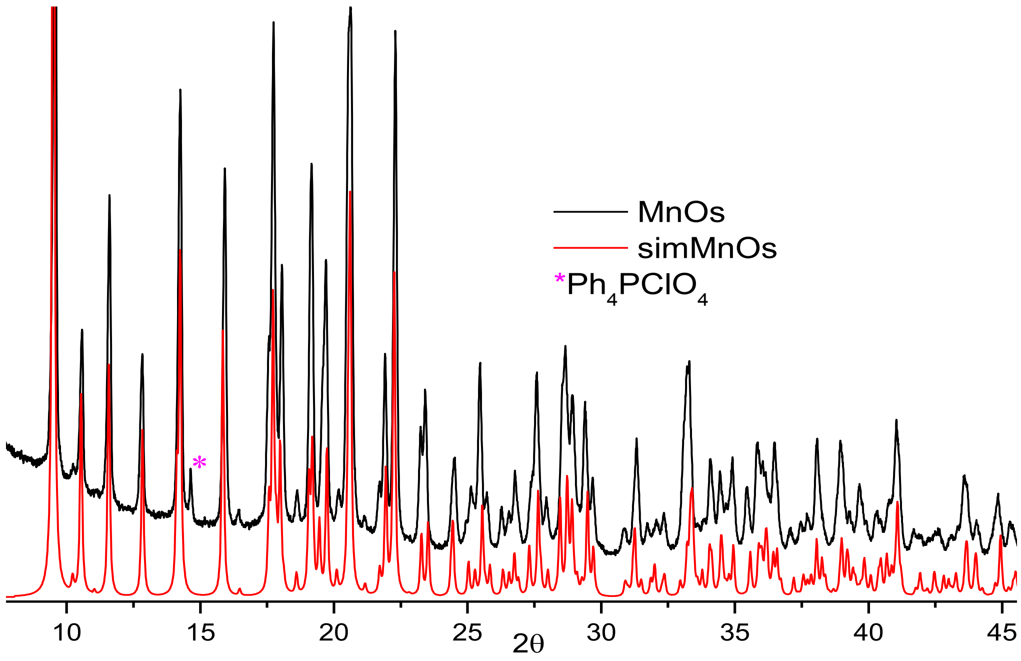

Figure S2. Simulated (red) and experimental X-ray powder pattern for neutral Mn-Os chain polymer.

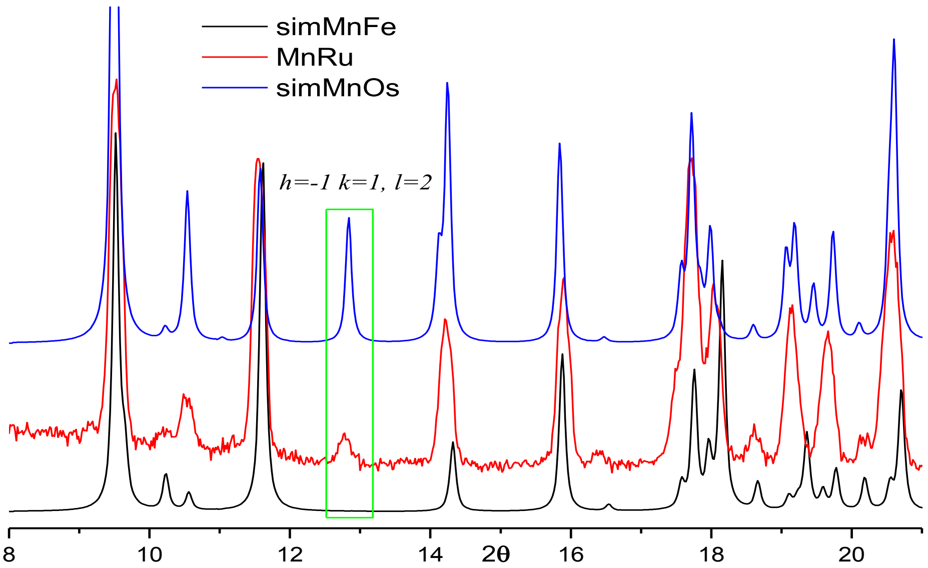

Figure S3. Additional reflections from (-1, 1, 2) plane consisted of metal atoms. Simulated (red) and experimental X-ray powder patterns for neutral Mn-Os chain polymer.

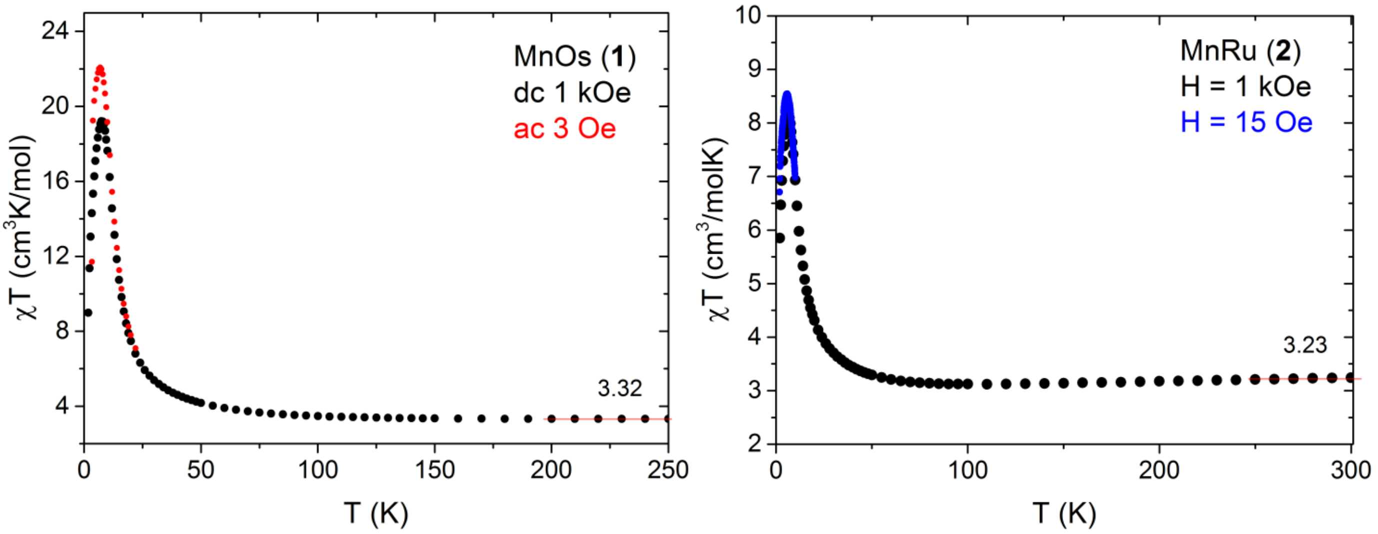

Figure S4. Magnetic susceptibility times temperature *vs* *T* at 1 kOe and lower fields for **1** in 3 Oe (left) and **2** in 15 Oe (right).

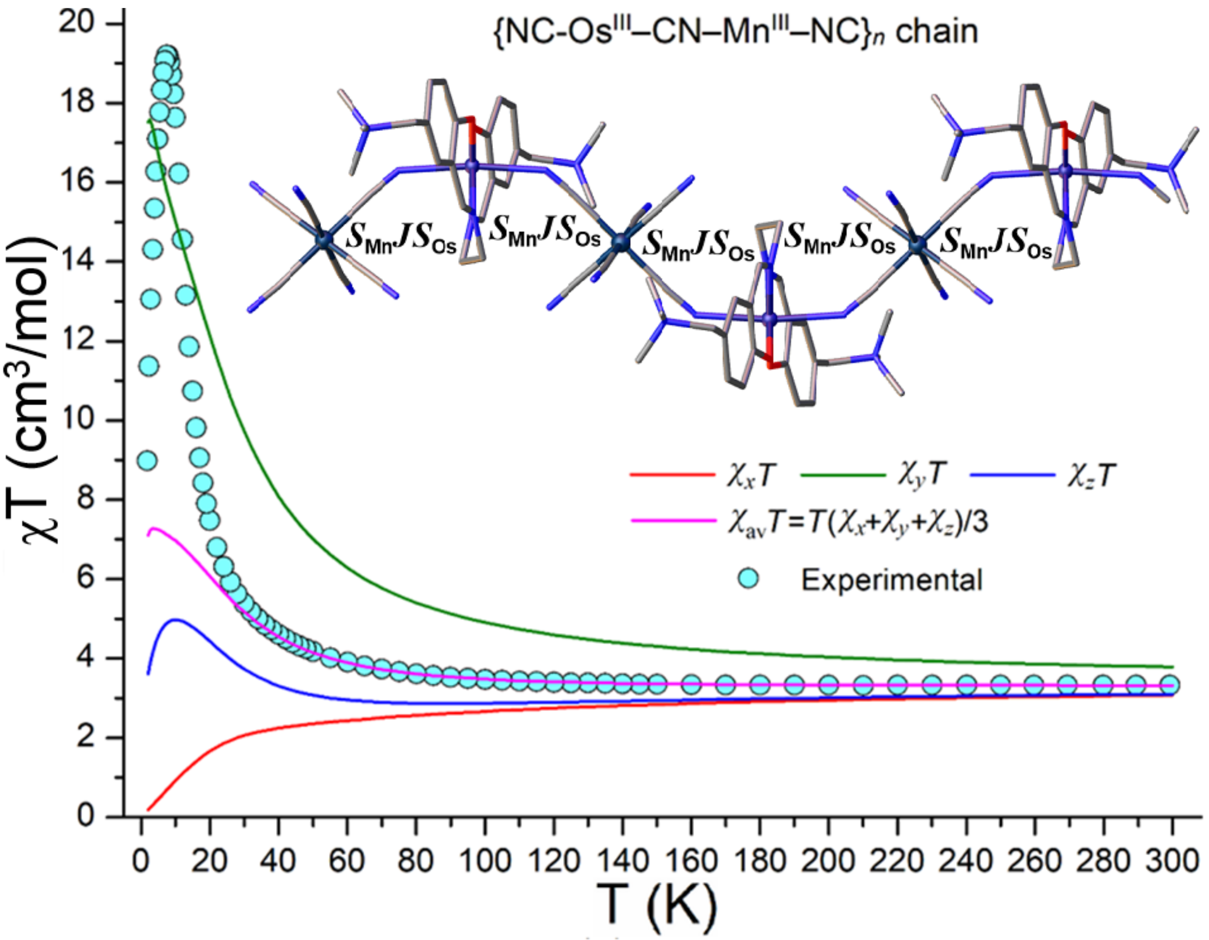

Figure S5. Calculated components χ_x, χ_y, χ_z of magnetic susceptibility of the Mn-Os chain (**1**). Below 50 K magnetic susceptibility is strongly anisotropic.

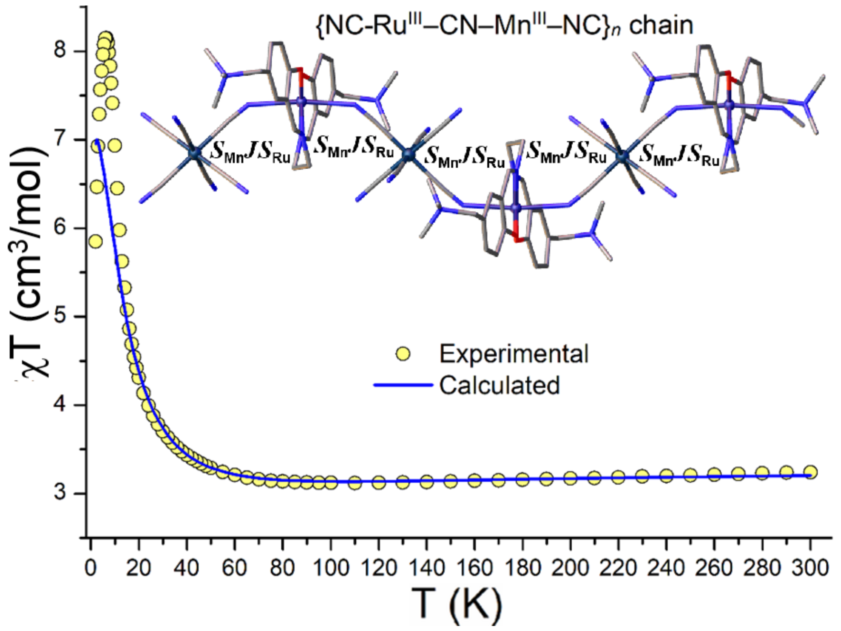

Figure S6. Experimental (yellow circles) and simulated (solid blue line) magnetic susceptibility χT of **2**. The χT curve was simulated with the spin Hamiltonian (Eqn. 2) involving anisotropic 3-axes spin coupling $\boldsymbol{S}_{\mathrm{Mn}}\boldsymbol{J}\boldsymbol{S}_{\mathrm{Ru}} = -J_x S_{\mathrm{Mn}}{}^x S_{\mathrm{Ru}}{}^x - J_y S_{\mathrm{Mn}}{}^y S_{\mathrm{Ru}}{}^y - J_z S_{\mathrm{Mn}}{}^z S_{\mathrm{Ru}}{}^z$. The best fit is obtained at the set of parameters $J_x = -18$, $J_y = +20$, $J_z = -18$ cm^{-1}, $g_{\mathrm{Mn}} = 2.00$, $g_{\mathrm{Ru}} = 1.80$, $D_{\mathrm{Mn}} = -4.0$ cm^{-1}. Calculations are performed for a six-membered fragment {Mn-Ru}$_3$ of the heterometallic chain of **2** with cyclic boundary conditions for the Mn-Ru spin coupling, as shown in the inset.

Calculations of magnetic susceptibility of chain compounds 1 and 2

Magnetic susceptibility of {Mn^{III}-Os^{III}}$_n$ (**1**) and {Mn^{III}-Ru^{III}}$_n$ (**2**) chain compounds are calculated in terms of a spin Hamiltonian

$$\hat{H} = \sum_{<ij>} \boldsymbol{S}_{\mathrm{Os}(i)} \boldsymbol{J} \boldsymbol{S}_{\mathrm{Mn}(j)} + \sum_j \boldsymbol{S}_{\mathrm{Mn}(j)} (\boldsymbol{T}_j(\theta) \boldsymbol{D}_j \boldsymbol{T}(\theta)_j^{-1}) \boldsymbol{S}_{\mathrm{Mn}(j)} + \mu_{\mathrm{B}} g_{\mathrm{Mn}} \boldsymbol{H} \sum_j \boldsymbol{S}_{\mathrm{Mn}(j)} + \mu_{\mathrm{B}} g_{\mathrm{Os}} \boldsymbol{H} \sum_i \boldsymbol{S}_{\mathrm{Os}(i)} \text{ , } \mathbf{(S1)}$$

where the sum <*ij*> runs over the neighboring Os(i) and Mn(j) cyanide-bridged exchange-coupled ions in the chain; the tensor of anisotropic spin coupling ($\boldsymbol{J}$) has a three-axes structure, $\boldsymbol{S}_{\mathrm{Mn}}\boldsymbol{J}\boldsymbol{S}_{\mathrm{Os}} = -J_xS_{\mathrm{Mn}}{}^xS_{\mathrm{Os}}{}^x - J_yS_{\mathrm{Mn}}{}^yS_{\mathrm{Os}}{}^y - J_zS_{\mathrm{Mn}}{}^zS_{\mathrm{Os}}{}^z$. The ZFS $\boldsymbol{D}_j$ tensors of the Mn^{III}(i) ions are supposed to have the axial structure (with $D < 0$ and $E = 0$). Each $\boldsymbol{D}_j$ tensor of Mn^{III}(i) ion is transformed by the $T_j(\theta)$ rotation matrix, $\boldsymbol{D}_j' = T_j(\theta)\boldsymbol{D}_jT_j(\theta)^{-1}$, specifying the non-collinear orientation ($\theta = \pm37.5°$) of these ZFS tensors in the bent structure of the $\{\text{Mn-Os}\}_n$ chain with respect to the local spin quantization axis z of the anisotropic exchange spin Hamiltonian $\boldsymbol{S}_{\mathrm{Mn}}\boldsymbol{J}\boldsymbol{S}_{\mathrm{Os}}$ (see inset in Figure 4). The components M_α ($\alpha = x, y, z$) of the magnetic moment $\boldsymbol{M}$ of vanadium complexes in an external magnetic field $\boldsymbol{H}$ are obtained from the conventional equation $M_\alpha = Nk_BT\dfrac{\partial \ln Z(\boldsymbol{H})}{\partial H_\alpha}$, **(S2)**,

where Z($\boldsymbol{H}$) is the partition function $Z(\boldsymbol{H}) = \sum_i \exp(-E_i(\boldsymbol{H})/k_BT)$, **(S3)**

with $E_i(\boldsymbol{H})$ being the energy of the i-th electronic state of the chain in the magnetic field $\boldsymbol{H}$ obtained from diagonalization of the spin Hamiltonian (S1). Then the diagonal component $\chi_{\alpha\alpha}$ of the tensor of magnetic susceptibility is written as $\chi_{\alpha\alpha} = M_\alpha/H_\alpha$; magnetic susceptibility of the powder sample is given by $\chi = (\chi_{xx} + \chi_{yy} + \chi_{zz})/3$. Calculations are performed at the experimental applied magnetic field of $H = 1$ kOe. With this approach, the temperature dependence of magnetic susceptibility χT of compounds **1** and **2** was calculated (Figures 4, S6 and S7).

Table S4. Cole-Cole fits parameters for **1**.

T (K)	τ (s)	$\Delta\tau$ (s)	α	Δα	X_0 (cm^{3}K/mol)	ΔX_0 (cm^{3}K/mol)	X_∞ (cm^{3}K/mol)	ΔX_∞ (cm^{3}K/mol)
2.80	8.41	0.43	0.4733	0.0026	7.70	0.13	0.1635	0.0022
2.95	2.359	0.060	0.4453	0.0023	7.283	0.061	0.1657	0.0029
3.10	0.629	0.015	0.3986	0.0039	6.527	0.052	0.1843	0.0070
3.25	0.2026	0.0037	0.3537	0.0049	6.044	0.037	0.205	0.011
3.45	0.05532	0.00089	0.3047	0.0059	5.635	0.027	0.225	0.016
3.65	0.01778	0.00026	0.2664	0.0060	5.344	0.019	0.247	0.018
3.85	0.006320	0.000087	0.2368	0.0058	5.116	0.014	0.264	0.020
4.10	0.002010	0.000029	0.2056	0.0062	4.874	0.011	0.290	0.026
4.30	0.000885	0.000013	0.1846	0.0062	4.7015	0.0089	0.319	0.031
4.65	0.0002622	0.0000048	0.1356	0.0061	4.4283	0.0051	0.524	0.042
5.00	0.0000938	0.0000025	0.0894	0.0053	4.1884	0.0021	0.780	0.061

Table S5. Cole-Cole fits parameters for **2**.

T (K)	τ (s)	$\Delta\tau$ (s)	α	Δα	X_0 (cm^{3}K/mol)	ΔX_0 (cm^{3}K/mol)	X_∞ (cm^{3}K/mol)	ΔX_∞ (cm^{3}K/mol)
2.60	0.1742	0.0061	0.5839	0.0042	3.461	0.028	0.1363	0.0083
2.80	0.0361	0.0015	0.5215	0.0087	3.064	0.028	0.190	0.019
3.00	0.01016	0.00043	0.449	0.012	2.774	0.021	0.267	0.026
3.20	0.00348	0.00016	0.388	0.013	2.574	0.015	0.325	0.030
3.40	0.001400	0.000058	0.326	0.012	2.4153	0.0096	0.401	0.030
3.60	0.000631	0.000024	0.266	0.012	2.2844	0.0062	0.482	0.029
3.80	0.000303	0.000011	0.213	0.011	2.1713	0.0039	0.558	0.031
4.00	0.0001576	0.0000067	0.162	0.010	2.0693	0.0024	0.635	0.035
4.20	0.0000934	0.0000048	0.1084	0.0100	1.9801	0.0014	0.760	0.041

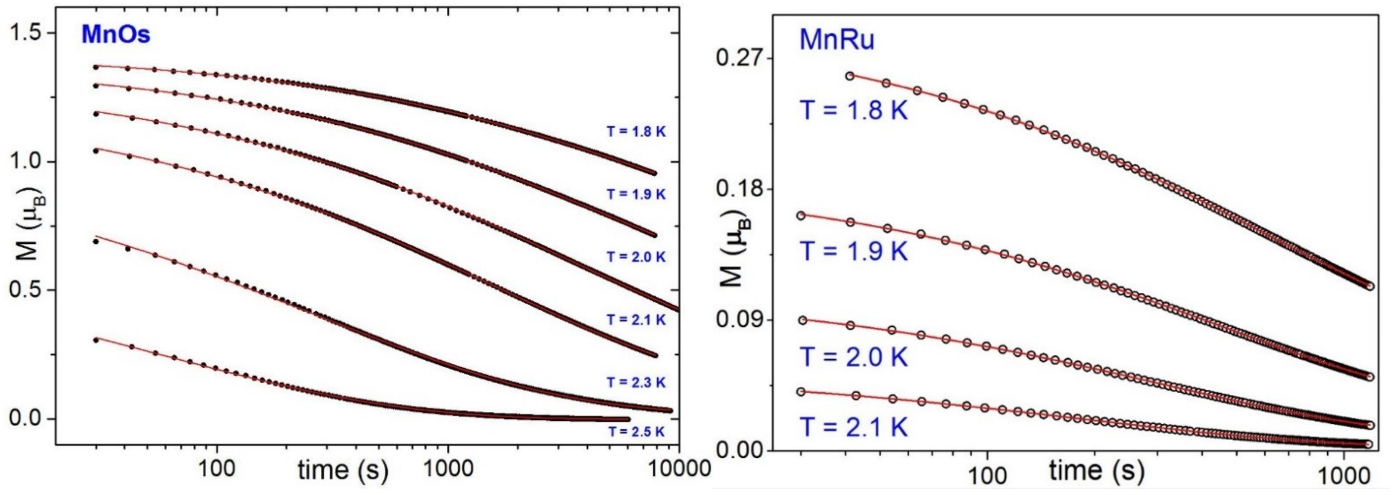

Figure S7. Time dependence of magnetization relaxation for **1** (left) and **2** (right) following the field change from 10 to 0 kOe, at constant temperatures 1.8 ÷ 2.5 K. The data were fitted using a stretched exponential decay: $M(t) = M_0 exp[-(t/\tau)^{1-n}]$. Only data for t > 80s were taken into account because it takes a finite time to switch off the field. In about 60 seconds, the field drops linearly from 20 to 0 kOe. The time when the current in the magnet reaches zero was defined as zero time in the above figures. It was confirmed by an attempt to fit a decay with the offset $(t - t_0)$, and values close to zero were obtained.

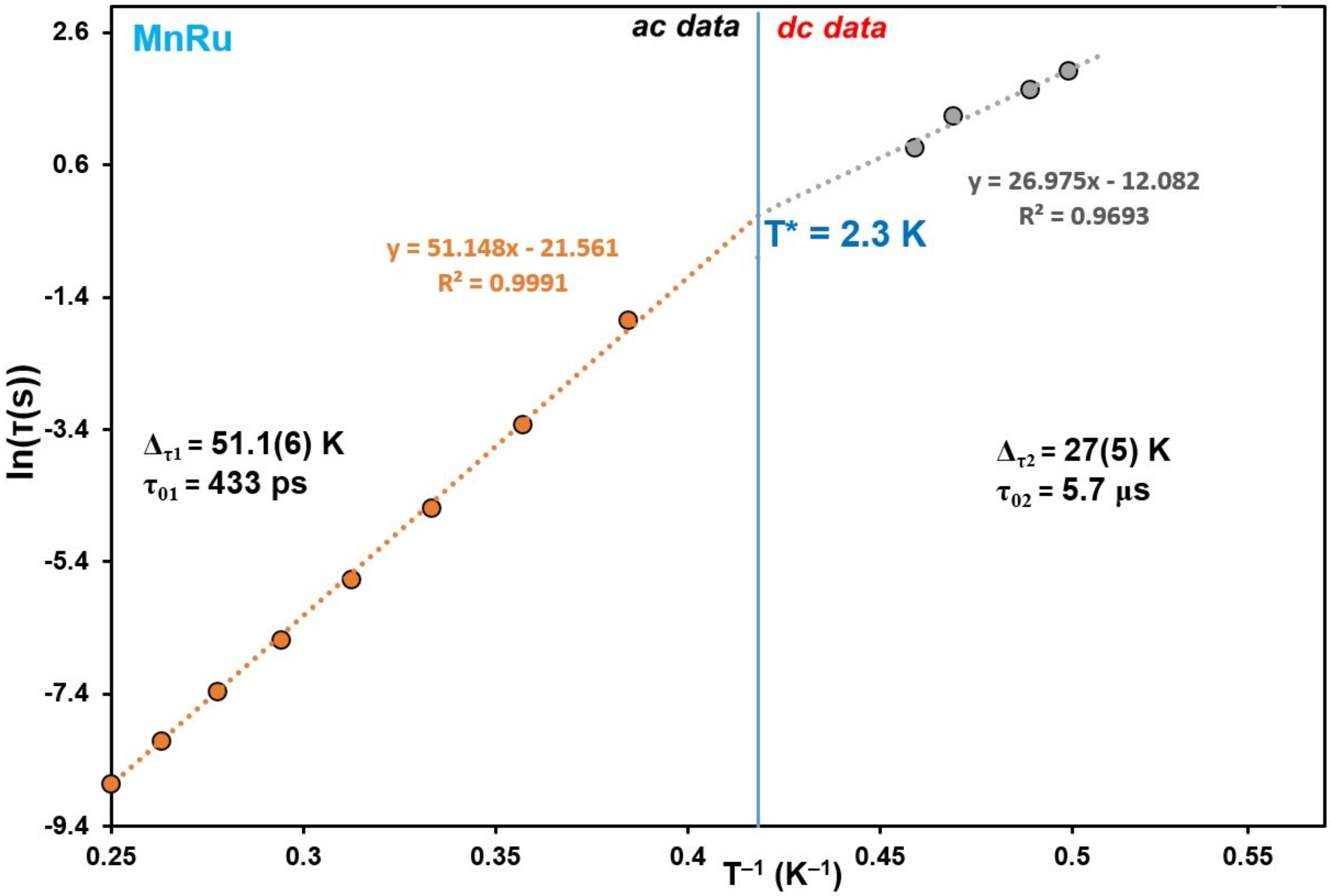

Figure S8. Relaxation time of **2** derived from the *ac* data (left) and time dependent *dc* magnetization (right). The dotted lines correspond to the linear fit according to the Arrhenius law: $\tau = \tau_{01} exp(\Delta_{\tau 1}/k_B T)$.

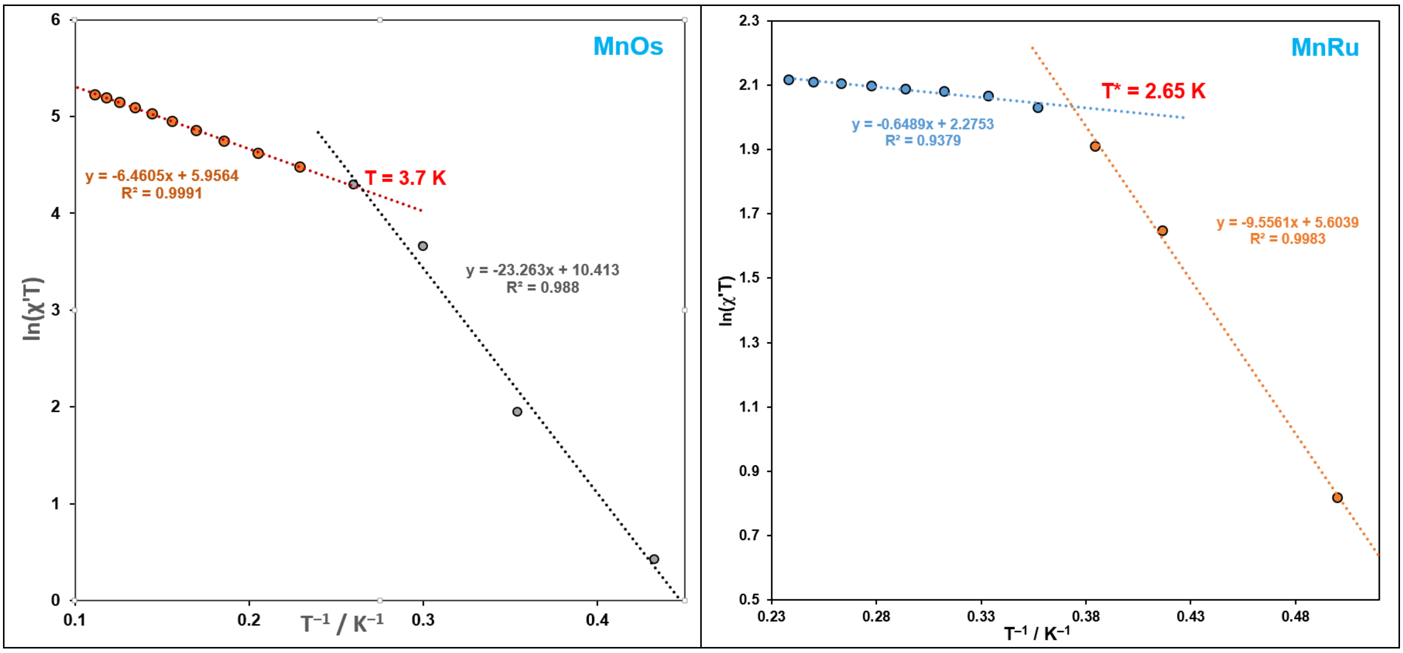

Figure S9. Crossover temperatures T^* obtained from the $\ln(\chi T)$ *vs* T^{-1} dependencies for the susceptibility data collected at 0 *dc* and 3 Oe *ac* field at 1 Hz below 9 and 4.2 K for **1** (left) and **2** (right) respectively.

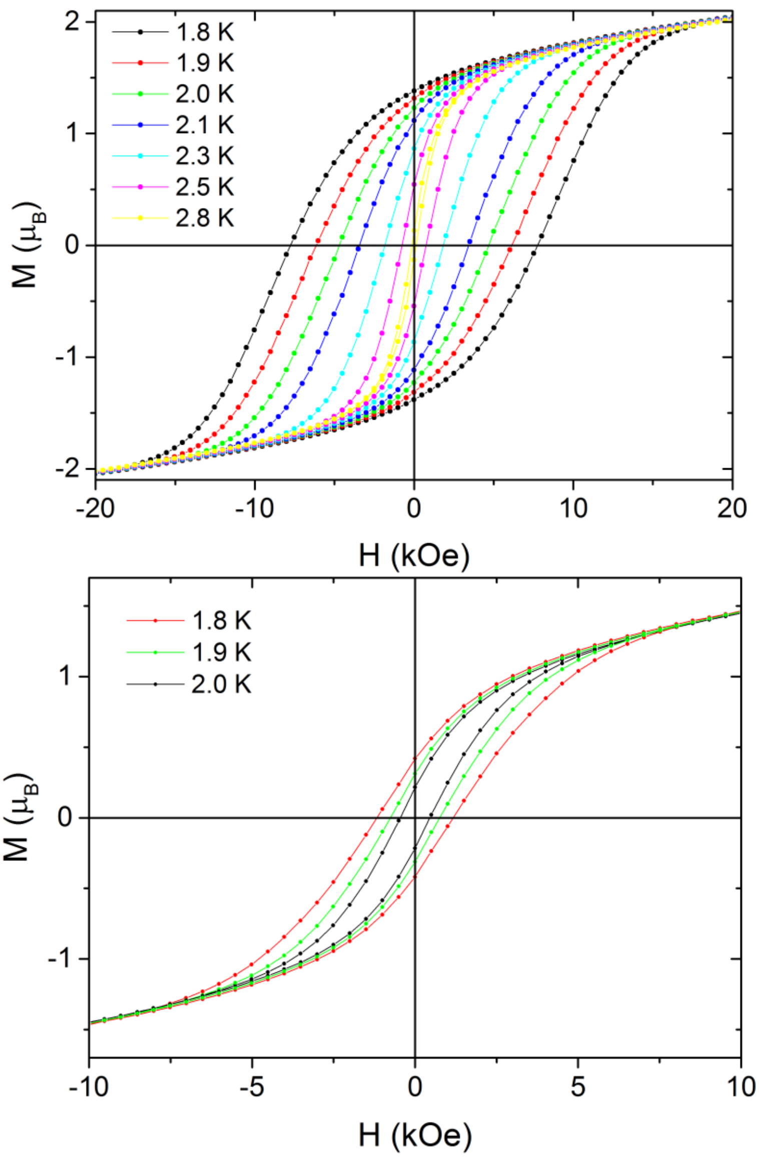

Figure S10. Magnetization versus field for **1** (top) and **2** (bottom) – hysteresis loops. Solid lines are to guide the eye.

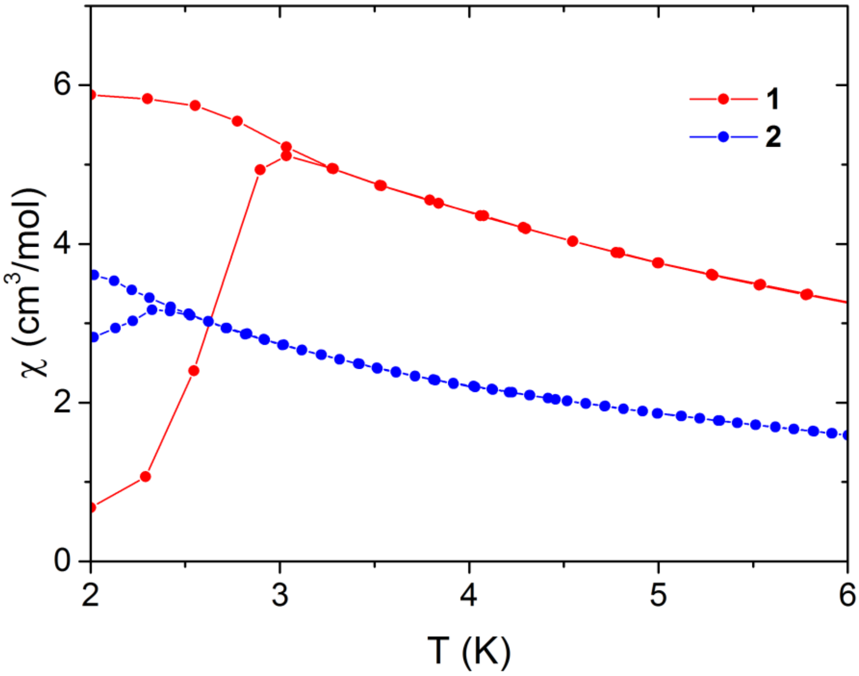

Figure S11. Zero-field cooling/field cooling magnetic susceptibility *vs.* temperature for **1** and **2** in 15 Oe with *a temperature sweep rate of 0.25 K/min up to 10 K and 2 K/min at higher temperatures for **1** and **2**.*

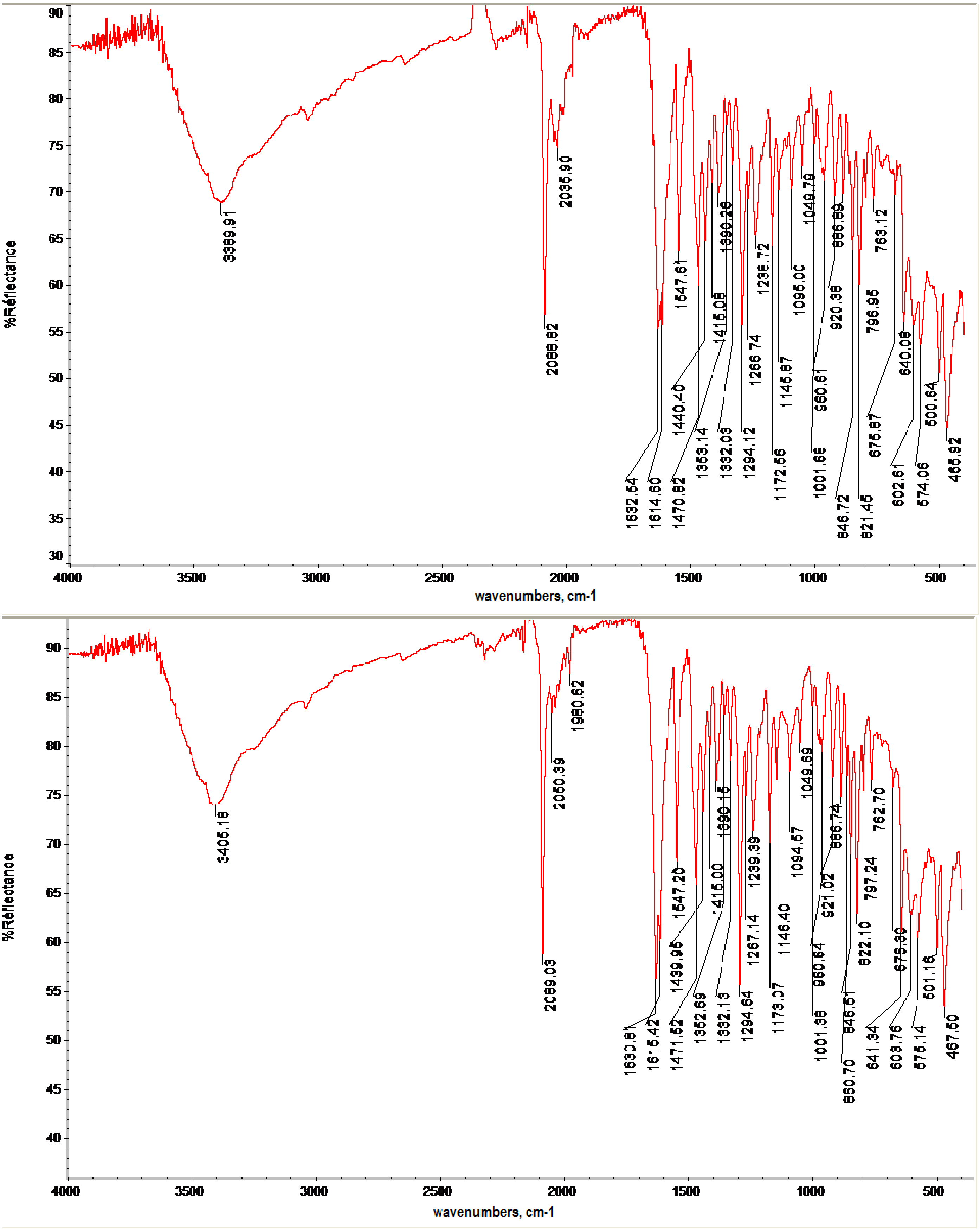

Figure S12. FTIR (ATR) spectra of **1** (top) and **2** (bottom).
